# A Systematic Review of Mapping Strategies for the Sonification of Physical Quantities

**DOI:** 10.1371/journal.pone.0082491

**Published:** 2013-12-17

**Authors:** Gaël Dubus, Roberto Bresin

**Affiliations:** Department of Speech, Music and Hearing, School of Computer Science and Communication, KTH Royal Institute of Technology, Stockholm, Sweden; University of Bath, United Kingdom

## Abstract

The field of sonification has progressed greatly over the past twenty years and currently constitutes an established area of research. This article aims at exploiting and organizing the knowledge accumulated in previous experimental studies to build a foundation for future sonification works. A systematic review of these studies may reveal trends in sonification design, and therefore support the development of design guidelines. To this end, we have reviewed and analyzed 179 scientific publications related to sonification of physical quantities. Using a bottom-up approach, we set up a list of conceptual dimensions belonging to both physical and auditory domains. Mappings used in the reviewed works were identified, forming a database of 495 entries. Frequency of use was analyzed among these conceptual dimensions as well as higher-level categories. Results confirm two hypotheses formulated in a preliminary study: pitch is by far the most used auditory dimension in sonification applications, and spatial auditory dimensions are almost exclusively used to sonify kinematic quantities. To detect successful as well as unsuccessful sonification strategies, assessment of mapping efficiency conducted in the reviewed works was considered. Results show that a proper evaluation of sonification mappings is performed only in a marginal proportion of publications. Additional aspects of the publication database were investigated: historical distribution of sonification works is presented, projects are classified according to their primary function, and the sonic material used in the auditory display is discussed. Finally, a mapping-based approach for characterizing sonification is proposed.

## Introduction

In this article we present a systematic review of results from studies in the field of sonification documented in 179 scientific publications representing 60 projects. The main idea is to draw an overview of a specific area of the relatively new research field of sonification in order to identify established methods and techniques. To this end, we have built up a database of sonification works currently comprising 734 entries. We set a particular focus on mappings of physical dimensions of sonified data to psychophysical and physical dimensions of the resulting sound, which we call auditory dimensions. We first present the concept of sonification: its nature, existing techniques, and a brief historical overview. In Section 2 we introduce our systematic review by presenting its objectives and restrictions. The method used for building the publication database and extracting the data is described in detail in Section 3. In Section 4 we present the sixty projects analyzed for this study by providing a brief description, mentioning the sonic material that was used, and listing the mappings. These data are analyzed and discussed in Section 5. The article ends with conclusions, suggestions for future work and the proposition of a mapping-based approach for characterizing sonification.

### 1.1 Nature of sonification

Several successive definitions of sonification have appeared since the concept was formally introduced in the 1990s. Although some earlier scientific works could qualify as genuine *sonification* (some are presented in Section 1.3), it seems that the term was first coined by William Buxton at a tutorial of the CHI conference in 1989, as:

*“The use of sound for data representation [, being] the auditory counterpart of data visualization.” [1]*

At first defined by analogy to scientific visualization, sonification rapidly gained significance as a research topic in itself, and the first conference dedicated to auditory display (International Conference on Auditory Display – ICAD) was founded in 1992 by Gregory Kramer. The numerous thoughts and findings resulting from this conference were summarized in *Auditory display* [2], published in 1994, where other definitions were proposed, the most elaborated being Scaletti's “working definition”:

*“A mapping of numerically represented relations in some domain under study to relations in an acoustic domain for the purposes of interpreting, understanding, or communicating relations in the domain under study.” [3]*

Sonification researchers gathered again at ICAD 1997 in order to report about the state-of-the-art of the field at that time and their ideas about future challenges. This led to the publication of the *NSF Sonification report* [4], where a new definition was formulated:

*“Sonification is defined as the use of nonspeech audio to convey information. More specifically, sonification is the transformation of data relations into perceived relations in an acoustic signal for the purposes of facilitating communication or interpretation.”*

The research community seems to have accepted this definition, as mentioned by Walker and Nees in the *Sonification handbook* [5] — the most recent effort to provide an exhaustive overview of the field.

Nevertheless, defining the boundaries of sonification is still a hot topic, with some researchers expressing the need of having a somewhat stricter, systematic definition [6], [7], whereas others are willing to step over the border to data-driven music [8]. This ambivalence is reflected in the ICAD program, where an increasing significance is attached to artistic works: a concert centered around sonification was included as a social event since 2000, a sonification contest has been organized since 2009, and topics of interest listed in the ICAD call for papers electively include references to art — “auditory display and art” in 2002, “sound as art” in 2010 and finally “sonification as art” in 2012 and 2013. Supper, in a sociological study of the ICAD community [9], reported the controversy created by Hermann's attempt to “*narrow down the boundaries of the field*”. Altogether, this janiform evolution indicates that a full consensus has not been adopted yet on the nature of sonification.

When comparing the successive definitions, it appears that qualifying a work as sonification is fundamentally related to its purpose: indeed, one could not determine if a sound is an emanation of sonification just by listening to it. This claim is in line with Scaletti's own reflexions on her early definition: “*That the sound be data-driven is necessary but not sufficient justification for calling it sonification; it must also have been done with the intent of understanding or communicating something about the original domain*” [3]. The significance of this aspect was recently supported by Varni et al. who claimed that, although not fitting into Hermann's definition, mapping to high-level auditory dimensions using music material should be allowed in sonification, provided that the main goal was “*to optimise efficiency of information communication*” and not “*o be pleasant to hear or to arouse particular feelings for the participants*”[10].

### 1.2 Character of sonification

The field of sonification is interdisciplinary by nature. Like visualization, it can be applied to any kind of data, interactively or not, making it potentially useful for a large set of different domains. Sonification as a research topic is itself at the junction of numerous scientific disciplines including human-computer interaction, psychoacoustics, engineering design, human factors and ergonomics, assistive technology, and cognitive sciences. This is nicely illustrated by the “nterdisciplinary circle of sonification and auditory display”in the introduction to the *Sonification handbook* [11].

As for any sort of auditory display, the use of sound as a medium for communicating information in sonification makes it particularly well suited for time-related tasks such as monitoring or synchronization. Taking advantage of the strong relationship between auditory perception and motor control [12], sonification can also be a valuable assistance to the perception of movements, and more specifically to the perception of one's own body motion, i.e. kinesthesia. Combining these two aspects makes sonification an ideal candidate to support the design of applications related to physical training and rehabilitation, e.g. in sport [13]–[16]. Other popular applications are in the fields of data exploration (e.g. [17]), data mining (e.g. [18]), and sensory substitution, e.g. for assisting visually impaired people [19], [20]. All in all, sonification represents a good complement to visualization insofar as the strengths of hearing and vision lie in different areas.

Various sonification techniques have been elaborated and formalized since the 1990s. The most widely accepted of these among the research community are described in detail in the *Sonification handbook*: audification [21], auditory icons [22], earcons [23], parameter mapping sonification [24], and model-based sonification [25]. *Audification* is the direct playback of data streams as sound waves, allowing only some minor processing for the signal to become audible. *Auditory icons* are based on an ecological approach to auditory perception, associating short environmental sounds with discrete events in the data in order to create metaphorical perceptual relationships, e.g. the mechanical “click” sound in digital cameras. *Earcons* are similar to auditory icons regarding how data are considered and with respect to brevity, but using entirely synthetic sounds with no prior metaphorical value, e.g. a melody indicating the battery level in mobile phones. Earcons create perceptual relationships that have to be learned from scratch, but can be easily parameterized and combined with each other to form hierarchical patterns of information.

*Parameter mapping sonification* consists in defining a set of mappings — the nature of which is discussed in Section 2.2 — between data dimensions and auditory dimensions. While simple to design, this technique has the potential to communicate information in a continuous manner, therefore being the most widely used sonification technique. Whereas it allows for a much greater flexibility than the previous techniques, the design of each mapping should, in return, be considered very carefully: an unfortunate choice can dramatically affect the usability of the whole system.

*Model-based sonification* was introduced by Hermann and Ritter [26] in an attempt to move away from the simplicity of parameter mapping sonification. Specifically designed for interactive contexts, model-based sonification aims at benefiting from the learning abilities pertaining to everyday listening [27], [28]. This technique is grounded in the human ability to associate a perceived sound and its characteristics with the source that generated it and its properties. For example we can distinguish between a broken table tennis ball and a new one by the different spectral characteristics of their impact sound, the sound of a broken ball having usually a higher centroid. Model-based sonification consists in defining a dynamic model representing a system that can evolve in time following a set of abstract laws, resulting in the creation of a virtual sounding object when data are injected into it. The sound is triggered when the user interacts with the system to activate the corresponding sounding object. The same model can be used with data coming from different domains, structurally different, and independently of their dimensionality. By analogy to the practice of a musical instrument, the model can be seen as a set of physical laws governing sound production and propagation, and the data as an instrument sounding only when manipulated by a player. Data from different domains could sound like different instruments, whereas structurally similar datasets would represent the same instrument with different qualities. To summarize, this approach allows the user to uncover relationships in the data in the same way that a musician would learn how to master an instrument.

### 1.3 Sonification in a historical perspective

History is rich with examples of use of the auditory modality to represent phenomena from the physical world. The use of auditing in Mesopotamia as early as 3500 BCE to detect anomalies in accounts of commodities is currently regarded as one of the first known implementations of data sonification [29]. Auditory displays have been exploited to perceive various physical dimensions such as temporal, physiological or kinematic variables long before sonification techniques were formalized: automatic alarm signals and striking clocks were used in ancient Greece (e.g. by combining a clepsydra with a water organ [30]) and medieval China to provide information about elapsed time. Pythagoreans reportedly defined a musical scale by associating different tones with various heavenly bodies according to their apparent velocity as seen from the Earth. Inspired by this approach in his treatise *Harmonices Mundi* (1619), Kepler transposed the Pythagorean concept of (harmony of the spheres) onto a heliocentric model. He assigned to each planet a fundamental tone depending on its aphelion (maximum distance to the sun), which was then changed in pitch depending on the angular displacement of the planet as seen from the sun, thus covering a specific interval as the planet moved around its orbit. This led him to focus on a harmonic relationship between the mean distance and the orbital period of a celestial body, which he finally discovered and exposed in his third law of planetary motion [31].

The stethoscope, a device performing the audification of heart rate, breath and blood pressure among others, was invented by Laënnec in 1816. Its design from the 1940s is still the one in use in everyday medical practice.

Probably one of the most well-known devices to integrate an auditory display system — popular among the public and emblematic for sonification researchers — is the Geiger counter, which translates ionizing radiation into clicks with a pulse depending on the level of radiation. But what made it so popular? This particular auditory feedback was originally designed as a complement to the visualization performed on the earliest devices by an electrometer, since this tedious method of measurement was not entirely satisfying. The first use of an auditory Geiger counter was reported in 1917, when a sensitive telephone was incorporated in the electrical circuit in order to listen to the audification of electrical impulses due to the ionization of the gas in the tube of the counter [32]. Already used nearly 40 years before for audifying a magnetically induced current in the nerves of frog legs [33] and in conducting wires subject to changes of molecular structure [34], this setup later evolved to include more advanced components for amplification and recording, loudspeakers, or headphones. By taking a step back and considering the Geiger counter as a device performing sonification of the level of ionizing radiation (instead of audification of electrical current), the issue of the mapping strategy emerges. Therein may lie the veritable key to its success: to transpose a physical quantity that is essentially non-visual — and pictured in everyone's imagination as very important because life-threatening — to the auditory modality through clicks with a varying pulse.

More recent applications of auditory displays were sparsely introduced during the twentieth century (e.g. Pollack and Ficks [35] in 1954, Speeth [36] in 1961, Kay [19] in 1974, Yeung [37] in 1980) but the starting point of the outburst of research in this field was the first ICAD conference in 1992 and the subsequent seminal work reported in the proceedings edited by Kramer [2]. Sonification, a particular case of auditory display, is therefore a relatively recent matter of concern for scientists, yet it has gained a certain maturity in about twenty years of research. Even if sonification is a narrow niche of interdisciplinary applied sciences — e.g. as compared to scientific visualization — the community of researchers has grown significantly and is now producing burgeoning examples of practical applications.

## Motivations

### 2.1 Why a systematic review?

We see the need for drawing an overview of the field of sonification in order to understand what the most successful and promising strategies are when sonifying data, and provide researchers, designers, and practitioners in the field with a starting point with strong foundations that will allow the field of sonification to make a leap forward. Our aim is to provide answers to questions such as: “What are the domains of application of sonification?”, “What is the historical distribution?”, “What kind of sound is used?”, and “What are the most popular mappings?”

More in detail, we want to organize the knowledge accumulated in nearly 20 years of research, learn from previous research which mappings are natural, popular, successful, or unsuccessful, and build a foundation for future sonification works. The aim of our study is to look at previous sonification designs in order to perform a systematic review of the mappings between physical and auditory dimensions present in the literature. We should be able to identify whether some particular associations between physical quantities and sound parameters are more used than others. This would not imply that these associations are the most successful ones, but it will suggest which should be investigated first, for example when designing new sonification-based applications.

We have therefore decided to focus on publications dealing with sonification, by combining results of a large number of independent studies. This will enable the identification of patterns in the published results, common trends, and critical sticking points. We developed a method for identifying potentially interesting papers, for extracting scientific information from them, and at the same time avoiding bias between articles providing very detailed descriptions and more concise ones (see Section 3). This resulted in a large pool of papers (about 700) that we organized into a database, from which we randomly selected sixty sonification projects for the systematic review introduced in Section 4. These projects correspond to a total of 179 scientific publications, and constitute a sample of typical sonification works.

There have been previous overview works in the field of sonification. The closest work to that described in the present study was documented in the pioneering work by Walker and Lane [38] who proposed the design of a database for providing “*a searchable online record of sonification mappings and auditory display techniques*”. Other overview works include a review of electronic aids for blind people [39], an overview of auditory display of molecular information [40], a review of biofeedback technologies for neuromotor rehabilitation [41], a study of evaluation methods for sonification [42], a study of sound synthesis tools used for sonification applications [43], a review of methods for image sonification [44], a historical review of the use of sonification in a database of networked music and sound art [45], a review of aesthetic strategies in sonification [46], a recent large review of visual, haptic, auditory and multimodal display [47], and overviews of the whole field of sonification (e.g. by Worrall [29] and in the *Sonification Handbook* [48]).

### 2.2 Mapping information to the auditory domain

In our study we define mapping in sonification as a function 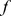 from a subspace 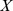 of the data domain to a subspace 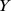 of the auditory domain. No condition is required on 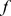, which can be non-linear, or even discontinuous. However, Scaletti [3] indicates that, in order to qualify as sonification, the mapping should not be completely arbitrary nor excessively complex (so that data relations are decipherable). The domain of 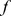, i.e. the data subspace 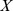, can be multidimensional, making 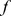 multivariate. In the case where elements of 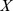 and 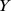 can be ordered, the mapping is said to have a polarity if 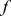 is monotonic.

Among the sonification techniques presented in the section 1.2, parameter mapping sonification is the most widely used for representing multidimensional data as sound. It is indeed the simplest way to continuously map data to sound. Using parameter mapping sonification forces the quantification of both the data subspace 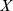 in input and the auditory domain subspace 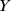 in output. Many dimensions can be displayed in parallel, and the mappings can be changed in real-time, making this method suitable for example in interactive sonification applications. Whereas they constitute the bulk of the design of a sonification system in the case of parameter mapping sonification, mappings can also be identified while using other sonification techniques.

As mentioned above, previous work on sonification mappings has been initiated by Walker et al., and is summarized in Walker's doctoral dissertation [49]. Walker and Lane's mapping database [38] was meant to organize sonification mappings according to three design components: nature, polarity, and scaling of the mapping (as documented in [50]). In doing so, they split up the design process of parameter mapping sonification into three stages: choice of the mapping strategy (i.e., which auditory dimension to use to represent a specific data dimension), choice of polarity, and psychophysical scaling. This work was based on perceptual studies to guide the design process following these three successive stages, and dealt with a limited number of generic data dimensions (e.g. “Temperature”, “Pressure”, “Velocity”).

### 2.3 Restriction to physical quantities

In the present work, we restrict our investigation to mappings between physical and auditory dimensions. We believe that we will come up with a list of mappings that could be easily implemented using physics-based sound models such as those provided in the Sound Design Toolkit [51]. This will allow for the design of a test bed for psychophysical experiments for the validation of the mappings and their properties, extending the pioneering study by Walker et al. [50].

We expect to extract a large variety of data from our database concerning domains, scales, and vocabulary. We will need to gather them into different categories at several levels, as presented in the next section. In this scope, the fact that physical quantities often correspond to concrete measures represents an advantage, the resulting categories being less subject to ambiguity than in the case of abstract data. Domains of mappings (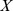 in Section 2.2) are subspaces of the considered data domain. Choosing the physical domain as data domain implies that the domains of mappings that we consider can most of the time be ordered (as these measures can be compared in the physical world). As a consequence, a polarity may be defined on this category of mappings.

## Methods

### 3.1 Building up the publication database

#### 3.1.1 Creating the publication database

We started our study by collecting a large pool of scientific publications in order to initiate the filling of the publication database.

Any type of work about sonification may include descriptions of mappings and may therefore be included in the publication database, provided that some part of the sonified data can be qualified as *physical quantities* as described previously. In practice, sonification projects are most often described with an acceptable depth in articles from peer-reviewed journals and conference proceedings, doctoral theses, and patent applications. Articles correspond to the most suitable format for our study: they are relatively short and describe often a single research project in a concise manner. We chose therefore to initiate the publication database by creating a pool of articles obtained by browsing several online journal databases (Springer Link [52], IEEE Xplore [53], ScienceDirect [54], PubMed [55], ACM Digital Library [56], ASA Digital Library [57], Ingentaconnect [58]), as well as proceedings from specialized conferences (ICAD [59], ISon [60], CHI [61], SMC [62], NIME [63], Audio Mostly [64]), and Google Scholar [65]. We do not, however, limit entries of the publication database to research articles, and other types of documents have been inserted following the expansion process presented in the next subsection. Doctoral theses can include unpublished project developments. Patents are by necessity technically more comprehensive than research articles, and can be helpful whenever the description in a related article is sparse or ambiguous. Some interesting information could also be extracted from book chapters, technical reports, master theses, artistic project descriptions, and websites, though those do not represent the majority of the target documents.

The first step of the article selection was performed by filtering the online databases listed above using the only keyword “*sonification*”, which typically gave a few hundred results in one go. Articles employing this term in the sense commonly used in biochemistry — i.e. sonic stimulation or irradiation by sound or ultrasound waves — were immediately discarded. We were aware that this process alone would not allow us to include projects published earlier than the formalization of auditory display techniques in the beginning of the 1990s, but this issue was later resolved by the process of expanding the publication database, as presented in the next subsection. For each search result, the criterion for inclusion in the publication database was the following: the title or the abstract of the article had to foreshadow the implementation of a practical application of sonification. It should not be too general like the presentation of a new software platform for sonification, nor too theoretical like the introduction of a taxonomy or a design framework. Sonification of abstract data such as stock market data or web traffic was left aside since we were only interested in physical quantities.

#### 3.1.2 Reading and expanding the publication database

With the method described in the previous subsection, we created an initial pool of articles, from which we could start our analysis. *Interesting* works cited in the articles from this initial pool were progressively included into our publication database. A given work was considered “interesting” whenever it matched the criterion for article inclusion defined in the previous subsection, i.e. the implementation of a practical application of sonification of physical quantities. This could be deduced either from the title and abstract as previously, or from the description in the citing article. In this way, the publication database could be expanded by including significant works published before the 1990s (i.e. before the term “sonification” appeared).

It appeared soon that reading and analyzing all the articles collected in the publication database would take a considerable amount of time, given that the number of entries seemed to grow exponentially, at least in a first phase. A shell script for the random selection of the next article to read was implemented in order to keep an even distribution of topics, research groups and time frame among the articles considered for the systematic review.

The systematic review is conducted on data extracted from *projects*, not from single articles. When an article is picked up from the publication database, we first look for *similar* articles stored in the publication database in order to group them into a project. Two articles are considered “similar” when they share the same objective, e.g. when the same data are used, or when new data are collected in a resembling experiment, using a resembling sonification algorithm. Similar articles are most of the time written by the same research group, have often the same funding source, and are usually published within a relatively short and homogeneous time frame. An article can form a project in itself when no similar articles can be found. More rarely, several projects can be tackled within a single article.

We tried to organize the reading of papers associated with a given project chronologically in order to follow and better understand developments and strategic choices. However, because of the backward-looking character of our searching strategy, we often found earlier references to be read at a later stage. These were either added to the group of articles associated with the project currently undergoing the reading process, or simply inserted into the publication database in case they belonged to another project.

The publication database was created according to the process described in the previous subsection in January 2011, encompassing therefore articles published in 2010 and before. In order to include more recent projects in the analysis, we repeated the creation process in January 2013 for articles published during the limited period 2011–2012. A total of 8 projects including one work from this period were included in the systematic review, representing 13.3% of the 60 projects. For future updates of the publication database, this operation can be reiterated for any period of interest.

Finally, review articles such as the ones mentioned in Section 2.1 allow both to include additional interesting references and to evaluate the progress state of the publication database.

### 3.2 Identifying mappings

#### 3.2.1 Criteria for mapping inclusion

Once the publication database was created according to the process presented above, all mappings of physical quantities to sound parameters were identified and considered for future analysis. However, some particular types of mappings were excluded from the analysis a priori.

In the beginning of the systematic review, we chose to consider audification as an absence of mapping, i.e. an absence of design strategy for the sonification system. Therefore we did not include works using audification in our publication database, and we did not count audification among the mappings to extract. This point of view was altered thereafter and we now believe that audification of data can be considered as a direct mapping of any data dimension to an elementary sound pressure level contributing to the creation of a waveform. Auditory graphs [66] were considered too abstract to be included in the analysis, as long as they did not correspond to a concrete sonification example (i.e. explicitly representing a given physical dimension). Although incorporating some data that can be classified as an objective physical dimension, e.g. the position of a cursor on a screen, auditory menus [67] were also judged too abstract to be included in this study.

Since the focus of the analysis was set on the design process of sonification systems, observations posterior to design (i.e. associations between physical and auditory dimensions that had not been consciously planned as part of the design but emerged from the use of the system as unexpected side-effects) were not considered as proper mappings. As an example inspired by a model-based sonification implemented by Sturm [68], one can consider a set of particles moving in a space subject to given physical laws of motion, each particle producing a pure tone of frequency depending on its velocity. An increase of temperature of the whole system would give rise to a higher perceived pitch of the sound feedback due to an increased overall velocity, but if the sonification design is not specifically mentioning the mapping Temperature 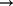 Pitch, the only one to be retained is Velocity 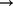 Pitch. Conversely, both should be taken into account if the intention of the sonification designer to make use of this particular behavior of the system is explicitly expressed at the design stage, even if the mapping is indirect. This example shows that this criterion is particularly relevant for model-based sonification, where the “sound-link variables” [69] (the dimensions of the model being directly sonified) are often acting on the sonic result at a low level.

#### 3.2.2 Mapping labels

An interesting aspect of the systematic review is the possibility to determine which mappings have been assessed as successful, or unsuccessful respectively. In order to track mapping evaluations performed in the considered different projects analyzed, we defined two corresponding labels: “assessed as good” (G) and “assessed as bad” (B). The label G was assigned to a given mapping whose efficiency was found to be significantly better when tested in comparison to other mappings corresponding to the sonification of the same physical dimension. The label B was assigned similarly when its efficiency was tested and found to be significantly worse than other mappings, or whenever a given mapping was reported inefficient for performing a given task. It is important to note here that we do not count the ability to perform a task as validation of the efficiency of a mapping if it has not been compared to another mapping. On the other hand, it seems reasonable to consider the inability to perform a task as a proof of its inefficiency.

Another label (F) was used to characterize mappings mentioned as interesting for a future application, but not implemented at the time the work was published.

#### 3.2.3 Classification process

**Figure 1:**
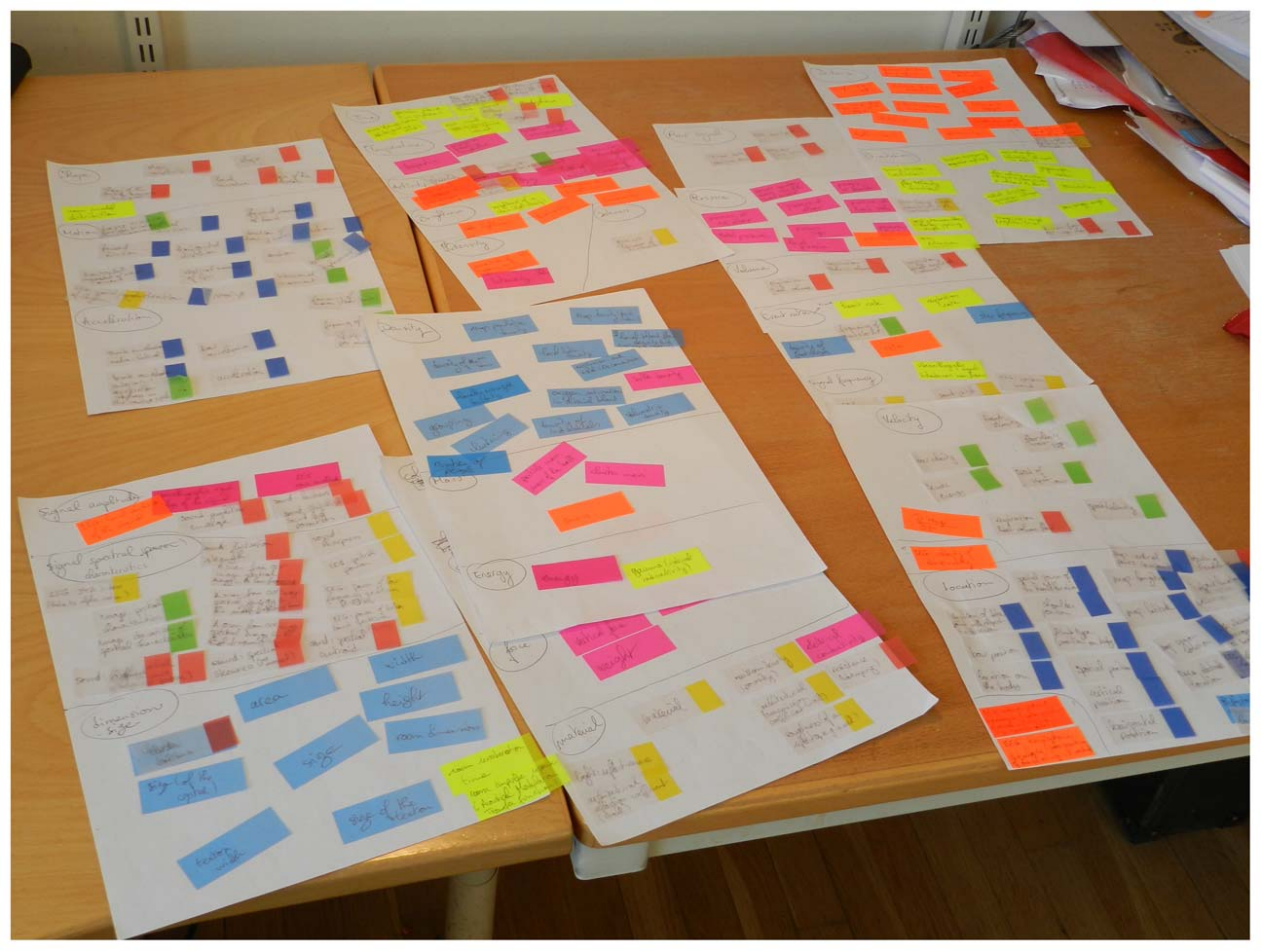
The classification was elaborated through a brainstorming using affinity diagrams. Each low-level dimension was written on a post-it note. The notes were then moved to form clusters based on their degree of similarity, constituting the intermediate-level dimensions used to reference mappings in this systematic review for both physical and auditory domains.

Due to the interdisciplinary nature of sonification, we expect many different types of physical quantities to be sonified. We conducted a classification process aimed at gathering similar data under intermediate-level conceptual dimensions. This process could be described as organizing chaotic information to form categories based on similarity and natural relationships. To this end, we used affinity diagrams, also known as the KJ method, a popular tool used in management and planning since the 1960s [70]. Physical dimensions directly extracted from the projects were written on post-it notes that were grouped by similarity on blank pages (Figure 1). Several clusters emerged and were assigned a label representing an intermediate-level conceptual dimension. For instance, the intermediate-level dimension Density encompasses the following lower-level variables: bulk density, population density, density of footsteps, number of people, local blood flow density level, oxygen saturation in arterial blood, water or forest density on a map, local data density, end tidal carbon dioxide concentration measured in respiration, density of He^++^ ions, neutron density reflecting material porosity, and spatial period of period-based textured images. The resulting classification, presented in Section 4, is inevitably based on the authors' interpretation of the data, and therefore incorporates elements of subjectivity. The intermediate-level dimensions were gathered into five high-level categories in order to reduce the dependency of the results to this subjectivity.

A similar issue occurs with auditory dimensions due to variations in the terminology. We used the same process in the auditory domain as for classifying the physical dimensions, gathering several words corresponding to the same notion in intermediate-level conceptual dimensions, which we grouped subsequently in five higher-level categories.

Arfib et al. [71], defining a theoretical framework for mapping gesture data to sound, make a clear distinction between dimensions corresponding to the perceptual effect on the listener (belonging to the “sound perceptual space”) and dimensions relative to “synthesis model parameters”. As mentioned in Section 1.1, sonification is indivisible from its purpose, which is to communicate information to a human user. We chose therefore to align our classification with the sound perceptual space, focusing on perceptual effects rather than on sound synthesis techniques. A simple illustration is the distinction between *Frequency* and *Pitch*. Whereas it is well-known that the two are directly related to each other [72], they belong to different spaces in the classification of Arfib et al., being respectively a synthesis model parameter and a dimension of the sound perceptual space. Sonification designers often employ the two terms alternately to describe a given mapping, as if interchangeable. According to our interpretation of those design descriptions, the resulting perceptual effect corresponding to the use of either word is most of the time identical. Following our perceptual approach, all the mappings concerned are classified as associating a specific physical dimension with Pitch. In some other cases, however, the distinction is clearly made by the sonification designer and is a well thought-out part of the design. For example, Grond and Dall'Antonia [73] map the distance between two atoms of a molecule to the center frequency of several superimposed resonant filters, which has the effect of modifying the timbre of an earcon rather than its pitch.

Various levels of description are expected to be found in the publication database, depending on the background and interests of the researchers designing the sonification system. For instance, the same mapping effect in the auditory dimension could be described rather approximately as a change in *Timbre*, more precisely as a change of *Brightness*, more specifically as an increase of the *Frequency of the spectral centroid*, more technically as an increase of the *Cutoff frequency of a bandpass filter* used to synthesize the sound, and so on. That being said, different levels of description can also reflect objective differences in the mapping design. To address this problem, the classification was built with enough flexibility to incorporate a multi-level hierarchy, taking into account the most detailed level of description available in the projects, for both physical and auditory dimensions.

Another source of disparity is the use of data sharing the same physical nature but on different scales. Gathering the dimensions according to their nature results in physical homogeneity, but also in having extremely different scales stirred together in the same dimension. One can wonder if it is pertinent to consider, for instance, temperature measurements in daily weather records as having the same significance as temperature measured inside a nuclear reactor, or core temperature of a star. The three variables described above belong to the same category of the current classification (Temperature) but could be distinguished at a lower level if the need for a finer distinction emerges in the future.

In light of the foregoing, it appears that the best solution is probably to have a multi-level and multi-scale structure for the classification of both physical and auditory dimensions. We provide an example of each in our current classification of auditory dimensions, further developed in Section 4.3: Spatialization incorporates a detailed multi-level set of subcategories, whereas Duration includes several scales. It should be noted that the aim of the present article is not to present a kind of ultimate classification, if that is ever possible. We shaped our classification by ensuring plasticity, i.e. the possibility to evolve dynamically to adapt to context changes. Context change here can correspond to the apparition of new data categories, or to a hitherto unprecedented discrimination of a data dimension according to different subgroups (e.g. scales). As it turned out, getting a better hindsight of the data resulted in the emergence of more stable categories. We believe that we have reached a relatively steady classification for auditory dimensions and high-level categories in the physical domain. Future developments of the classification could be validated by confrontation with the opinion of researchers from diverse fields, for example using a distributed and cooperative version of the KJ method [74] over the World Wide Web, or by using coding schemes [75]. The latter, combined with inter-coder reliability tests, could yield a more robust validation of current and future classifications.

## Presentation of the Data

### 4.1 Publication database

The publication database currently comprises 739 entries [76]. Sixty projects were analyzed, corresponding to 179 publications referenced in the present article, and are presented in Section 4.4. The remaining 560 entries of the database, selected as potentially interesting but not analyzed yet, can be browsed online.

### 4.2 Sonified physical dimensions

In the domain of sonification mappings, i.e. sonified physical quantities, 33 dimensions emerged from the classification process and are presented in Table 1 along with a label (from P01 to P33) for reference in the remainder of the article. These dimensions, whose names are self-explanatory, are distributed over five high-level categories: kinematics, kinetics, matter, time, and dimensions. *Kinematics* refers to quantities used to characterize motion and position. *Kinetics* refers to quantities linked to the causes of motion, and by extension those related to energy. *Matter* refers to properties of the matter. *Time* refers to characteristics of a signal in the time-frequency domain. *Dimensions* refers to geometry of objects and spaces.

**Table 1:** Intermediate-level conceptual dimensions in the physical domain.

Label	Physical dimension	Category
P01	Location	Kinematics
P02	Velocity	
P03	Acceleration	
P04	Jerkiness	
P05	Distance	
P06	Orientation	
P07	Motion	
P08	Energy	Kinetics
P09	Intensity	
P10	Force	
P11	Temperature	
P12	Activity	
P13	Pressure	
P14	Signal amplitude	
P15	Material	Matter
P16	Density	
P17	Mass	
P18	Transmission characteristics	
P19	Reflection characteristics	
P20	Roughness	
P21	Color hue	
P22	Color saturation	
P23	Color luminosity	
P24	Time elapsed	Time
P25	Phase	
P26	Event rate	
P27	Signal frequency	
P28	Signal spectral energy distribution	
P29	Volume	Dimensions
P30	Size	
P31	Area	
P32	Length	
P33	Shape	

List of physical dimensions sonified in the articles from the publication database, arranged according to their corresponding high-level category.

### 4.3 Auditory dimensions used in sonification

In the codomain of sonification mappings, i.e. sound parameters, 30 dimensions emerged from the classification process and are presented in Table 2 along with a label (from A01 to A30) for reference in the remainder of the article. These dimensions are distributed over five high-level categories: *Pitch-related*, *Timbral*, *Loudness-related*, *Spatial*, and *Temporal*. Six dimensions belong to at least two high-level categories. Most of the names of the dimensions are self-explanatory. In the following we describe those requiring further clarification.

**Table 2:** Intermediate-level conceptual dimensions in the auditory domain.

Label	Auditory dimension	Category
A01	Pitch	Pitch-related
A02	Pitch range	
A03	Timbre	Timbral
A04	Instrumentation	
A05	Polyphonic content	
A06	Voice gender	
A07	Allophone	
A08	Spectral power	
A09	Amplitude of harmonic	
A10	Frequency of harmonic	
A11	Roughness	
A12	Brightness	
A13	Center frequency of filter	
A14	Saliency	
A15	Loudness	Loudness-related
A16	Dynamic loudness	
A17	Spatialization	Spatial
⋅ 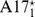	Stereo panning	
⋅ 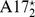	Multichannel panning	
⋅ 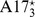	Vector base amplitude panning	
⋅ 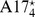	Head-related transfer function	
⋅ 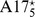	Ambisonics	
⋅ 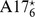	Interaural time difference	
⋅ 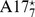	Interaural amplitude difference	
⋅ 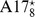	Interaural frequency difference	
⋅ 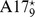	Non-specified spatialization method	
A18	Doppler effect	
A19	Tempo	Temporal
A20	Duration	
⋅ A20_1_	Rhythmic duration	
⋅ A20_2_	Event duration	
⋅ A20_3_	Ambient duration	
⋅ A20_4_	Non-specified duration scale	
A21	Sequential position	
A22	Melody lead	
A23	Articulation	
A24	Decay time	
A25	Melody	Pitch-related, Temporal
A26	Harmony	Pitch-related, Timbral
A27	Chord progression	Pitch-related, Timbral, Temporal
A28	Spectral duration	Timbral, Temporal
A29	Reverberation time	Spatial, Temporal
A30	Performance activity level	Loudness-related, Temporal

List of auditory dimensions used for sonification in the articles from the publication database, arranged according to their corresponding high-level category. Dimensions belonging to more than one high-level category are displayed at the bottom of the table. The multi-class dimension Spatialization is distinguished from the others using a star (*) in its label, which also incorporates an index differentiating the different subclasses. Similarly, the label of the multi-scale dimension Duration incorporates an index differenciating the different scales.

Timbre is usually defined as comprising all the characteristics allowing us to distinguish between two sounds having identical pitch and loudness. Because this is a negative definition, it often appears judicious to describe a mapping more specifically than using the term “timbre”. The role of the high-level category *Timbral* (labels A03 to A14) is to cover this usual definition of timbre, whereas the intermediate-level dimension called Timbre (A03) in our classification corresponds to all the cases where no further precision on the mapping was given. Instrumentation (A04) refers to cases where musical instruments change depending on the sonified data, whereas Polyphonic content (A05) refers to the number of parts in a polyphonic piece, i.e. the number of instruments rendered in the playback of the piece. Spectral power (A08) encompasses operations performed on the sound spectrum that are not covered by the remaining dimensions listed in the high-level category *Timbral*.

Spatialization (A17) corresponds to the position of a sound source in space and time. Since it is often described through several interrelated aspects, it constitutes a good illustration of multi-level classification. These aspects include equipment (e.g. binaural earphones, stereo loudspeakers, array of loudspeakers), technique (e.g. Ambisonics, Vector Base Amplitude Panning, Wave Field Synthesis), as well as quantities centered on the perceptual effect on the listener (e.g. Interaural amplitude difference, Interaural time difference, Interaural frequency difference), or involving both technical and perceptual aspects (e.g. Head-related transfer function). While enumerated in Table 3, the different aspects are considered as a single mapping of the particular physical dimension to Spatialization. This applies even to cases where different aspects result in divergent assessments of efficiency, materialized by different mapping labels as described in Section 3.2.2. For instance, one could map the orientation of the listener towards a sounding object either to Interaural Time Difference (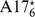) or Interaural Amplitude Difference (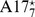) with varying success. However, from the point of view of the sonification designer, the goal remains unchanged: it is to map Orientation (P06) to Spatialization (A17).

**Table 3:** Description of the projects analyzed in this study.

Project number	List of publications	Summary of the work from a sonification perspective	Sonic material	Mapping references
*continued from previous page*				
1	[86]–[89]	Interactive sonification of the motion of aquarium fishes and ants	MIDI protocol, digital synthesizers, samplers, piece of music, Max/MSP [90]	P01 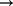 A01, P01 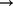 A04, P01 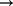 A12, P01 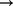 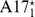 , P02 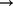 A19, P02 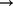 A20 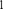 , P03 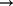 A19, P05 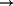 A15, P07 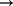 A12, P07 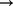 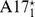 , P12 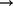 A04, P12 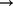 A15, P12 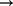 A19, P16 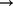 A04, P16 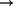 A15, P16 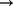 A25, P19 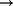 A03, P19 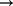 A04, P21 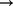 A01, P21 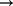 A03, P21 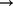 A04, P21 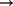 A06, P30 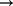 A01, P30 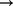 A03, P30 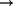 A04, P30 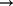 A06, P30 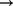 A25, P33 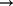 A03, P33 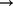 A04, P33 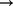 A06, P33 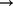 A25
2	[78], [91]	Model for the sonification of large datasets with application to seismic data	MIDI protocol, Sound Blaster MIDI synthesizer, environmental sounds	P01 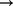 A01, P01 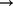 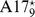 , P01 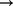 A20_1_, P01 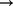 A20_2_, P05 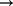 A20_1_, P05 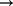 A28, P07 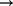 A20_1_, P07 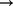 A28, P12 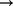 A20_1_
3	[92]	Sonification of acoustic and audio data	Various stimuli including filtered noise, pure and complex tones, and clicks	P14 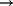 A01, P14 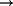 A15 ( 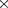 3), P18 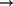 A20_1_, P19 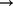 A15, P19 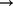 A20_1_, P25 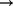 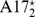 , P25 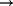 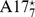 , P26 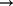 A20_1_, P27 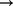 A01, P28 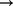 A01, P28 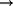 A02 ( 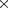 2), P28 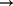 A11, P28 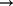 A12, P30 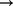 A01 ( 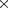 2), P30 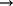 A08, P30 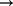 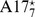 , P30 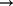 A20_1_, P30 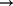 A20_2_, P30 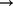 A28, P33 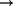 A01, P33 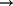 A26
4	[93]–[104]	Interactive sonification of colored images and video clips for the design of a mobility aid	Spatialized instrument sounds	P01 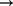 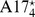 , P01 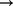 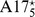 , P15 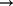 A15, P05 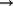 A20_1_, P05 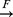 A29, P06 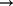 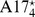 , P06 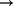 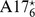 , P06 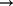 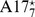 , P21 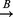 A01, P21 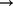 A03, P21 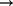 A04, P22 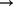 A01, P23 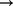 A01, P23 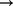 A04, P23 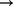 A15
5	[105]–[110]	Sonification of video clips of counter-movement jumps for studying multimodal integration	Synthesized voice, amplitude and frequency modulated tone	P10 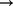 A01, P10 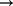 A15
6	[111]–[114]	Interactive sonification as a help for positioning surging instruments, offline sonification of human EEG as a tool for analysis of long recordings	Samples and environmental sounds modulated in pitch, volume and balance	P01 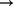 A01, P01 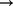 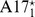 , P07 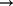 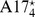 , P07 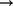 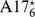 , P07 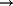 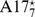 , P07 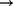 A20_2_, P07 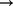 A28, P28 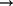 A01, P33 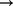 A28
7	[115]	Art installation: an immersive virtual world using interactive sonification	Filtered noise bursts, wide-band signal, subtractive synthesis instruments	P01 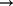 A01, P01 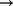 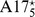 , P16 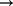 A01, P16 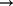 A08, P16 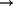 A20_1_, P16 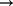 A20_3_
8	[116]	Sonification of contour maps	Sampled piano tones	P01 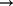 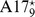 , P05 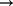 A01, P05 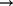 A02, P05 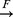 A15, P32 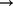 A01, P32 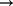 A02, P33 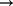 A12, P33 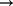 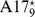
9	[117]–[128]	Interactive sonification of rowing for elite and visually impaired athletes, extended to recreational sporting activities	Pure tone, triangular wave tone, MIDI protocol, sampled xylophone tones, piece of music, vocal formant synthesis, bandpass-filtered noise synthesizing the sound of wind, PureData [129]	P03 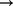 A01 ( 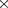 2), P03 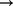 A07, P03 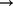 A12, P03 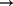 A15, P04 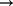 A12, P26 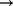 A19
10	[130]	Sonification of meteorological data (hail storms)	FM instruments, FM synthesis	P01 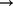 A01, P01 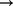 A03, P01 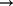 A15, P01 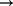 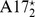 , P01 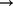 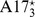 , P01 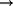 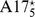 , P30 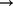 A15, P30 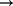 A20_2_
11	[131], [132]	Sonification of geophysical maps	MIDI synthesizer	P06 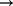 A01, P06 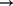 A03, P06 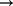 A04, P06 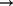 A19, P06 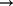 A20_1_, P11 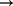 A01, P16 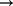 A01, P16 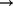 A03, P16 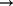 A04, P16 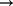 A19, P16 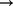 A20_1_, P24 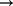 A01, P24 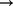 A03, P24 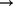 A04, P24 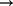 A19, P24 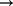 A20_1_
12	[17], [133]	Sonification of well logs	Granular synthesis, timbre grains for musical instruments, Geiger counter metaphor	P06 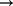 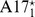 , P06 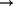 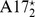 , P08 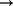 A01, P08 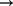 A20_2_, P08 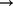 A28, P14 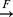 A20_4_, P15 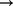 A03, P16 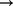 A01 ( 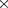 2), P16 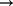 A20_2_ ( 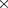 2), P16 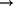 A28 ( 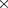 2), P18 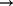 A01, P18 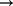 A20_2_, P18 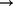 A28
13	[134]–[137]	Interactive sonification of: activity in social spaces, motion of a calf, movements of a violin player, and free gestures	MIDI protocol, Max/MSP	P01 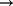 A01, P01 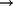 A03, P01 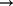 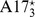 , P02 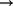 A01, P02 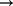 A19, P03 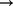 A01, P03 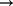 A03, P03 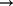 A15, P05 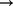 A01, P06 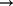 A01, P07 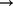 A01, P07 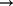 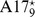 , P07 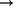 A18, P09 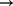 A16, P12 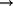 A03, P12 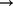 A14, P12 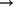 A15, P12 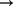 A26, P16 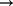 A03, P16 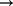 A16, P16 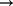 A20_2_
14	[138]–[141]	Sonification of textured MRI images	Synthesized speech-like sounds	P06 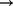 A21, P16 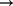 A01, P16 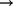 A03, P16 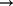 A08, P16 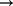 A20_1_, P16 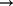 A28, P28 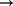 A08 ( 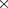 2), P30 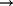 A08, P30 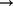 A20_1_, P32 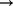 A20_1_, P33 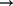 A08
15	[50], [142]–[148]	Psychoacoustical study of sonification mapping strategies	Pure tones, FM synthesis	P02 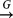 A01, P02 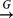 A12, P02 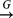 A19, P05 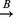 A01, P05 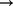 A12, P05 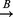 A19, P11 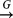 A01, P11 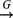 A12, P11 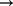 A15, P11 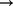 A19, P11 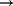 A23, P13 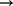 A01, P13 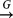 A12, P13 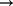 A15, P13 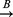 A19, P13 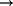 A23, P17 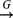 A01, P17 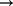 A12, P17 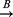 A19, P26 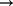 A01, P26 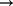 A15, P26 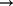 A19, P26 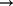 A23, P30 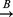 A01, P30 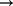 A12, P30 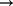 A15, P30 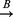 A19, P30 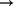 A23
16	[149]–[152]	Sonification of geospatial data with uncertainty	MIDI protocol, sampled piano and trumpet tones	P01 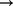 A01, P05 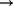 A01, P11 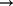 A01 ( 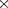 2), P32 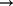 A01
17	[153]	Interactive navigation in a virtual space containing auditory targets	Songs	P05 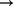 A15, P07 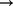 A18
18	[154], [155]	Interactive sonification of running mechanics	Environmental sounds, pre-recorded speech	P07 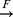 A08, P07 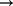 A15, P08 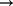 A15, P26 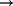 A15
19	[156]–[158]	Spectral mapping for real-time sonification of human EEG	Pure tones, coupled oscillators	P01 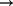 A01, P01 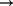 A02, P01 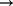 A04, P01 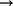 A12, P01 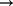 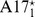 , P01 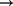 A21, P05 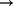 A01, P05 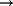 A15, P25 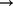 A03, P25 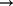 A11, P27 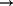 A04, P28 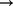 A01 ( 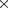 3), P28 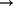 A08 ( 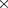 2), P28 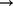 A15 ( 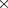 3), P28 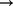 A20 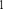 , P28 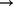 A28
20	[159]–[164]	Real-time event-based sonification of human EEG	Blip oscillator with vibrato, harmonic tones modulated with a percussive envelope, synthesis from pink noise grains	P01 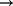 A01, P01 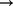 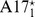 , P01 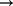 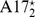 , P07 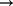 A03, P12 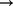 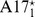 , P12 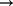 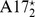 , P14 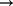 A15, P25 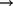 A12, P27 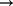 A01, P27 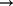 A04, P27 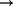 A12, P27 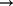 A20_1_
21	[165], [166]	Kernel regression mapping for real-time sonification of human EEG	Subtractive synthesizer for simple speech-like sounds	P01 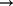 A10 ( 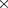 2), P02 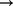 A01, P05 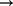 A08, P05 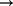 A14, P05 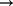 A15, P06 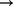 A10, P12 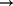 A07 ( 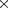 2), P12 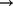 A10 ( 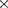 3), P26 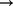 A09
22	[167]–[169]	Brain-computer interface for paralyzed patients, with real-time multimodal feedback	MIDI protocol, sampled piano tones	P14 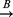 A01
23	[169]–[171]	Real-time orchestral sonification of EEG, breath, heart beat, ECG with application to artistic performances	MIDI protocol, sampled tones of several instruments	P01 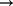 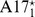 , P14 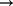 A01, P14 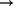 A15, P27 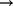 A01, P27 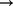 A04, P27 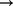 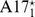 , P27 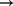 A20_4_, P28 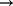 A01, P28 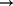 A15
24	[172]	Sonification of maps	Environmental sounds	P01 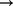 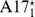 , P01 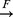 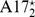 , P05 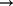 A15, P16 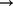 A15
25	[173]	Sonification of solar wind data	Synthesized wind sound, sampled vocal tones, pure tone, triangular wave tone, sawtooth wave tone, Max/MSP	P02 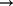 A12, P11 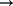 A01, P11 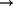 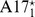 , P16 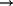 A15, P25 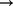 A01, P25 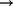 A12
26	[174], [175]	Interactive sonification of range measured with a laser pointer for mobility aid	MIDI protocol, QTMA software synthesizer, sampled piano tones	P02 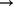 A01, P02 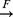 A15, P05 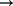 A01, P05 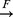 A15
27	[176]–[178]	Psychoacoustical study of semantic perception of non-musical rhythms and pitch changes in short non-speech sounds for earcon design and sonification of emotional and directional content	MIDI protocol, sampled vibraphone and recorder tones	P02 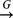 A19, P02 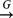 A20_1_, P07 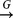 A01, P07 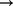 A01, P07 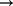 A15 ( 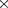 2), P07 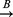 A19, P07 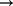 A19 ( 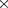 2), P07 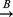 A20 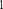 ( 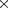 2), P07 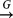 A25, P07 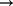 A25, P09 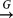 A19, P09 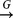 A20_1_, P12 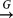 A19, P12 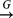 A20_1_
28	[179]–[197]	Real-time sonification of physiological data (pulse oximetry, respiration, blood pressure) for anaesthesia monitoring, hypothetical sonification support for landing multi-engine aircraft	“*Beeps”*, “*breathy”* tone (bandpass-filtered white noise, abandoned), “*relatively pure”* tone (with few harmonics), earcons and beacons (“*tones*”)	P01 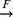 A21, P02 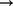 A12, P02 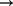 A16, P02 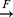 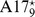 , P02 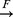 A19, P06 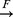 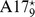 , P08 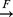 A01, P13 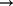 A01, P13 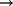 A20_1_, P16 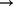 A01 ( 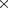 2), P24 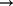 A20_2_, P26 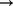 A19 ( 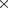 2), P29 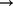 A12, P29 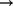 A15, P29 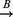 A20_1_, P32 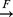 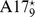
29	[198]	Multi-level interactive sonification of single molecule properties based on force spectroscopy data	Oscillators, IIR filters, pitched tones	P10 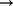 A01, P10 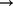 A15
30	[199]–[203]	Interactive sonification of trunk kinematics for balance improvement	Pure tone	P03 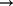 A01, P03 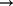 A15 ( 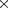 2), P03 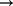 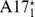
31	[204], [205]	Sonification of knee-joint VAG signal	Pure tone	P14 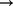 A15, P27 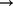 A01
32	[206]	Model-based sonification for audiovisual composition and performance: evolutionary model of a swarm populated with virtual agents evolving according to a genetic algorithm	Primitive DSP units from SuperCollider (generators and processors). Each agent possesses its own sonic signature.	P01 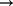 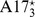 , P02 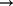 A15, P05 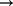 A29, P07 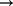 A18, P26 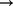 A01
33	[207]	Sonification of OCT images of human tissue for discrimination of tumor and adipose	FM synthesis	P06 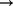 A01, P28 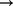 A08, P28 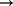 A12, P28 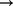 A15
34	[208], [209]	Interactive sonification of violin bowing	Percussive “*click*” sound, pitch-modulated “*interesting waveform*”, oscillator	P01 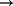 A01 ( 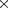 2), P02 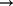 A01, P03 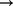 A01, P06 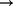 A01, P07 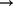 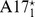 , P26 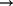 A15, P26 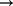 A20_1_
35	[26], [69]	Model-based sonification of high-dimensional data sets: particle trajectories moving in a data potential	Additive synthesis	P12 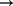 A15, P17 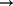 A01 ( 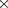 2), P18 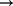 A24, P30 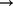 A01 ( 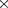 2)
36	[26], [69], [210], [211]	Model-based sonification of high-dimensional data sets: data solid constituted by point masses anchored via springs, thus vibrating, dynamically evolving following a growing neural gas algorithm	Additive synthesis, damped linear oscillators	P01 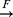 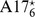 , P08 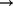 A15 ( 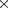 2), P16 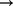 A01
37	[212]	Model-based sonification of high-dimensional data sets using principal curve as time-trajectory	Time-variant oscillators, Geiger counter metaphor: ticking sound synthesized by an exponentially decreasing sine wave	P01 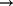 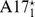 , P02 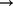 A15, P05 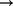 A15, P07 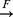 A18, P16 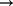 A01, P16 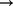 A20_4_, P33 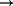 A01
38	[213]	Model-based sonification of high-dimensional data sets based on a crystallization process of points in a Euclidian vector space	Additive synthesis, time-variant sine oscillator	P08 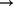 A15, P30 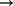 A01, P33 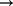 A12
39	[214]–[217]	Real-time sonification of EMG signals	Six sine oscillators with constant frequency carrier between 200 and 1600 Hz, set in harmonic relationship and modulated in amplitude	P01 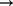 A01, P01 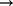 A02, P12 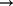 A03, P14 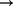 A09, P24 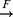 A11
40	[218]	Auditory display of complex graphical objects, e.g. color images, based on speech annotations and sonification of colors	Not specified: “*[It] can be artificially generated sounds, sounds of musical instruments, or other sounds*”	P21 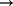 A01, P21 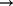 A03, P22 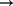 A15
41	[219]	Interactive sonification of segmented two-dimensional images for the purpose of gaining spatial awareness of the image structure	VST instruments (abandoned), FM synthesis, AM synthesis, filters, square wave tone	P01 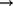 A01, P01 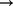 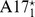 , P05 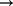 A13, P05 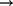 A20_4_, P07 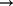 A01, P07 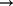 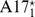 , P07 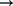 A25, P24 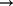 A13, P31 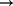 A01, P32 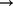 A11, P32 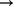 A15, P33 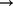 A02, P33 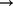 A19, P33 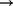 A25
42	[220]–[222]	Interactive audiovisual biofeedback system for stroke rehabilitation	Musical sounds, MIDI protocol, sampler, Max/MSP	P01 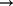 A01, P01 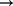 A02 ( 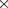 2), P01 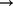 A03, P01 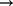 A04 ( 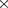 2), P01 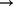 A08, P01 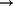 A15, P01 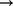 A20_1_ ( 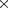 2), P01 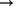 A26, P02 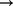 A20_1_, P04 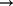 A20_1_, P05 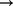 A27, P07 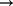 A27, P25 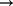 A27
43	[19], [223]–[227]	Mobility aid for the blind using ultrasonic echolocation, first monaural (Sonic Torch) then binaural (Sonic Glasses), enabling the detection of objects and their perceptualization within a range of 0 to 6 m	Echo of an ultrasonic sinusoidal wave modulated in frequency by a sawtooth wave, heterodyned with the original signal and rescaled to audible frequency (resulting in practice in a pitched tone) interrupted by short silences (10% of the time). This method was first implemented with pulses instead of frequency modulation, and was found to be much less successful.	P05 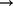 A01, P06 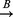 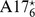 , P06 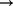 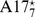 , P06 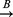 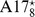 , P20 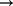 A08
44	[228]	Device converting words on a computer to phonemes via brightness level in order to translate contemporary news headlines to dadaist poems	Phonemes from SpeakJet synthesizer IC	P23 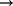 A08
45	[229]	Interactive sonification of three physiological quantities for artistic performance (“*biomusic*”)	Max/MSP, OSC protocol, filter-enhanced audification, unvoiced subtractive synthesis (filtered noise, flange effect), voiced subtractive synthesis (sawtooth band-limited signal)	P11 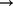 A13, P11 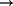 A15, P29 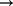 A01, P29 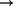 A13 ( 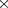 2), P29 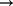 A15 ( 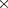 2)
46	[230]	Interactive sonification of facial movements and expressions	PureData, cosine wave oscillator, sweeping filter, sampled sounds, additive synthesis	P07 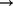 A01 ( 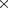 2), P07 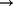 A12
47	[73]	Sonification utility for molecules (SUMO) illustrated by the case of amino acids and B-factors	SuperCollider, OSC protocol, resonant filters (Klank, Formlet) implementing earcons	P01 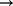 A01, P01 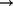 A13, P01 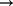 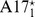 , P01 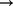 A21, P05 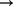 A13, P05 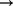 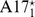 , P05 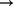 A21
48	[231]	Interactive sonification of diverse information related to a mobile device (smartphone), including a model of virtual balls anchored via springs, bouncing inside a box	Sample banks of different impact sounds between various materials	P01 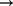 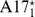 , P08 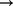 A15, P17 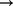 A01
49	[232], [233]	Interactive sonification of CFD simulations for computational steering	Max/MSP, filtered white and pink noise to simulate the sound of wind, combinations of pure tones	P01 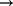 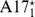 ( 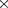 2), P02 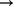 A01, P02 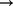 A12, P02 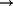 A13, P02 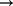 A15, P05 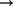 A01, P05 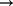 A15, P06 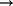 A15, P06 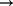 A26
50	[20], [234]–[236]	iSonic: a tool for interactive sonification of georeferenced data on choropleth maps for visually impaired users	Java MIDI sounds: instruments (strings, piano) playing scales, spatial sound server for use of generic HRTF, prerecorded samples	P01 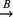 A01, P01 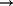 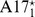 , P01 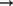 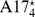 , P01 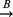 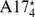 , P01 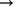 A21, P16 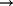 A01, P26 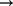 A20_4_
51	[237]	Navigation assistance system on a PDA for visually impaired	Not specified for the sonification part	P05 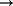 A01, P05 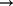 A15, P05 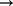 A19, P06 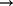 A01 ( 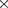 2), P06 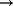 A15 ( 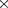 2), P06 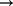 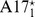 , P06 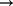 A19 ( 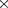 2)
52	[15]	Six methods for interactive sonification of water pressure changes in the context of crawl stroke swimming	Five sine oscillators with nine semitones of pitch range, white noise modulated in amplitude fed into subtractive synthesis, formant filter synthesis, additive synthesis controlled by a low-frequency pulse	P01 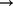 A02, P01 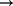 A10, P13 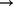 A01 ( 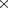 3), P13 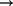 A09, P13 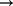 A12 ( 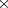 2), P13 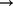 A13, P13 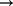 A15 ( 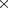 2), P13 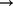 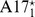 ( 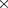 2), P13 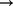 A20_1_
53	[238]	Interactive sonification of radial direction	MIDI software synthesizer	P06 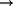 A07
54	[10]	Interactive sonification of the synchronization of two subjects performing hand gestures with mobile devices, three different sonification methods	Multitrack music audio file, Moog ladder filter, MIDI music file, Permorfer (program for real-time modification of music performance)	P08 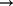 A15, P08 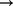 A19, P08 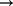 A22, P08 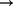 A23, P08 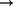 A30, P25 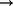 A05, P25 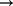 A12, P25 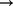 A22
55	[239]	Sonification of star brightness data with focus on aesthetics for use in music	MIDI protocol, audified data modulated in amplitude by other data	P23 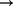 A01, P23 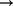 A15
56	[240]	Interactive sonification of navigation through geocontextual maps for visually impaired people using mobile devices	PureData, processed pre-recorded human speech	P01 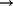 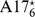 , P01 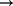 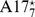 , P05 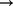 A15, P05 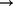 A20_1_, P05 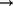 A21, P05 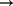 A29, P06 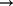 A13, P06 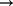 A21
57	[241]	Sonification of video data showing the movements of a worm	Max/MSP, granular synthesis, short sine tone wavelets grains	P01 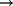 A01, P01 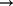 A23, P01 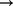 A28, P23 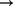 A15
58	[242]–[250]	The Cosmophone: art installation performing real-time sonification of the trajectory of cosmic particles (muons)	Two arrays of loudspeakers (below, above) for spatial sound, MIDI protocol, MIDI synthesizer, Max/MSP, samples of rain drops, piano tones and scattered words from a poem	P01 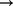 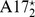 , P07 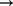 A03, P07 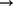 A15, P07 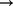 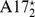 , P07 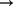 A18, P07 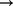 A29, P08 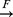 A01
59	[251], [252]	The Sonified Urban Masterplan (SUM): a tool for sonification of urban maps, evolved to a tool for computer-aided composition of graphic scores via path-based image sonification	PWGL (Lisp-based visual programming environment for music), MIDI protocol, OSC protocol, possibility to interface in Lisp, Max/MSP and PureData	P01 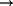 A01, P16 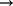 A15, P21 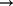 A03, P23 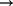 A15, P32 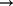 A01, P32 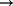 A20_4_
60	[253]–[256]	Interactive sonification of body movements (tilt of hips and torso) using the wearable interface hipDisk	Twelve simplistic tones generated by a Basic Stomp2 microcontroller forming a one-octave chromatic, pentatonic, major or minor scale	P06 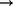 A01

Description of the projects analyzed in this study including their corresponding list of publications, sonic material used, and identified mappings between physical (P) and auditory (A) dimensions. Mappings are referenced using the labels defined in Tables 1 and 2. Mapping labels (G, B, F) described in Section 3.2.2 are displayed over the mapping arrows. Multiple occurrences of the same mapping identified within the same project are indicated in parenthesis following the concerned mapping reference. Abbreviations used in this table are listed in Table 4.

Tempo (A19) should be understood in accordance with its definition for the MIDI format: it represents a high-level control on the playback speed independent of the density of sound events.

Duration (A20) corresponds to distance on the time axis, i.e. the time elapsed between two events. The quantity usually referred to as “tone duration” corresponds to the time elapsed between a tone onset and its offset. Another quantity commonly used in the study of music performance is Inter-Onset Interval (IOI), defined as the time elapsed between two successive tone onsets. Tone duration and IOI are therefore included in the same category. The reason for not distinguishing between these two quantities in our classification originates in the lack of precision found in many publications describing mappings, often mentioning the “duration” of sound stimuli without specifying clearly if it corresponds to tone duration or IOI.

Duration was chosen to illustrate the multi-scale classification due to the dependency of the perception of duration to time scale. According to Sethares [77], sonic events occurring at different time scales activate different cognitive structures calling on different types of memory (echoic, short-term, or long-term memory). This disparity of perceptual impressions was taken into account by Saue [78], who selected four time scales to be used in the context of sonification of large datasets: spectral, rhythmic, event, and ambient. These time scales were used to derive four elements of our classification.

The first element based on time scale is Spectral duration, corresponding to the smallest time scale (less than 50 ms). Sethares describes how echoic memory operates at this scale, performing the “*fusion*” of sonic events into coherent cognitive structures such as pitch and timbre. For his part, Saue explains that these sonic events are perceived as “*variations in timbre and localization*”. For reasons exposed above, in our bottom-up approach to classify auditory dimensions, timbre is often presented indirectly by sonification designers. Timbre variations are often described through performing low-level manipulation of the signal, or assembling temporal elements belonging to the spectral time scale (e.g. grain duration in granular synthesis). On the other hand, both spatialization and pitch were found to be described more explicitly by sonification designers. As a consequence, Spectral duration was represented by an auditory dimension in itself (A28) belonging to the high-level categories *Temporal* (by essence) and *Timbral* (by design).

The three other elements based on time scale constitute the multi-scale dimension Duration (A20): Rhythmic duration (A20_1_) corresponds to a duration comprised between 100 ms and 2 s, calling on short-term memory, and described by Saue as “*perceived as relative changes to events inside auditory streams*”. Event duration (A20_2_) corresponds to a duration over 2 s, calling on long-term memory, and described by Saue as “*perceived as irregularly spaced singular events*” and by Sethares as “*disconnected events*”. Ambient duration (A20_3_) refers to dynamic continuous auditory streams that are not perceived as events but, according to Saue, “*as always present (or not perceived at all); a state of no-change or slow change*”. Finally, similarly to the case of a multi-class dimension, a last element has to be added to handle cases where no specific scale is mentioned (A20_4_).

### 4.4 Description of the projects

The sixty projects analyzed in this systematic review are presented in Table 3. For each project, we provide a brief description of the work through the prism of sonification, focusing on this particular aspect rather than on the own research questions of the researchers. Interactivity, an important characteristic of a sonification system that has been highlighted by Hunt and Hermann [79], was characterized by the use of the words “interactive” and “real-time” in the description. We also describe the sonic material that was used, ranging from detailed descriptions of the sound synthesis to software and hardware platforms, in order to get a sense of the tools used by sonification researchers, a concern that was shared by Bearman and Brown in their recent review study [43]. Finally, the list of mappings identified according to the process described in Section 3.2 is displayed. The list of abbreviations used in Table 3 is presented in Table 4.

**Table 4:** List of abbreviations used in Table 3.

AM	Amplitude Modulation
CFD	Computational Fluid Dynamics
DSP	Digital Sound Processing
ECG	Electrocardiography
EEG	Electroencephalography
EMG	Electromyography
FM	Frequency Modulation
HRTF	Head-Related Transfer Function
IC	Integrated Circuit
IIR	Infinite Impulse Response
MIDI	Musical Instruments Digital Interface
MRI	Magnetic Resonance Imaging
OCT	Optical Coherence Tomography
OSC	Open Sound Control
PDA	Personal Digital Assistant
QTMA	QuickTime Music Architecture
VAG	Vibroarthrographic
VST	Virtual Studio Technology

List of abbreviations used in Table 3.

## Results and Discussion

### 5.1 Mapping frequencies

#### 5.1.1 Expectations

The principal measure considered in the systematic review is the frequency of use of mappings. In a preliminary study [80], we formulated three assumptions to be verified for a larger number of sonification projects. These assumptions, based on 54 publications representing 21 projects, constitute our preliminary hypotheses concerning sonification mappings and are summarized hereunder.

##### Hypothesis 1

A large proportion of sonification mappings follow the logic of ecological perception. Mappings are often performing a sort of simulation of underlying physical phenomena, which can be implemented either directly or metaphorically. These natural associations between sound and its meaning regarding physics were called “*universal relationships*” by Hermann and Ritter [69] and depicted as “*deeply engrained in the way we* — *usually subconsciously* — *pick up meaning from sound events*”.

##### Hypothesis 2

Pitch is by far the most used auditory dimension in sonification mappings. Typically, the design process for a sonification system often starts by mapping the most important data dimension to the frequency of a pure tone — it is, as Henkelmann puts it [81], the “Hello World” of sonification. Pitch is known to be the most salient attribute in a musical sound, described as “*the most characteristic property of [musical] tones, both simple (sinusoidal) and complex*” [82] and “*the most common dimension for creating a system of musical elements.*” [83]. Although creating a sonification system is not equivalent to composing music, sonification designers are certainly influenced by music, its structural forms, and its aesthetic values [84], [85].

##### Hypothesis 3

Spatial auditory dimensions are almost exclusively used to sonify kinematic physical quantities.

In the remainder of this section we introduce several methods for investigating these hypotheses, as well as some other aspects of the data extracted from the publication database.

#### 5.1.2 Census of mapping occurrences

A total of 495 occurrences of mappings were identified within the 60 projects analyzed. In order to determine the most popular mappings, i.e. those occurring the greatest number of times, we first performed a simple census by counting all the occurrences identified in this systematic review, which are listed in Table 3. We could then establish a ranking of the most used mappings, the fourteen most popular of them being presented in Table 5.

**Table 5:** Most used mappings within the sixty projects analyzed.

Number of occurrences	Mapping	Reference
24	Location 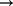 Spatialization	P01 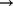 A17
18	Location 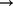 Pitch	P01 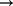 A01
12	Distance 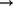 Loudness	P05 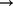 A15
10	Density 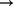 Pitch	P16 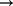 A01
9	Distance 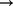 Pitch	P05 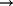 A01
8	Density 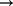 Duration	P16 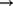 A20
7	Orientation 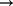 Pitch	P06 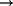 A01
7	Size 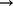 Pitch	P30 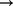 A01
6	Velocity 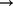 Pitch	P02 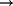 A01
6	Motion 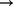 Pitch	P07 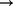 A01
6	Motion 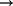 Spatialization	P07 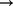 A17
6	Energy 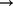 Loudness	P08 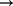 A15
6	Signal amplitude 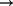 Loudness	P14 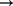 A15
6	Signal spectral energy distribution 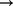 Pitch	P28 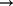 A01

The fourteen most used mappings within the sixty projects analyzed. More than half of these mappings involve Pitch (A01). All the other (i.e. not involving Pitch) mappings but one correspond to natural perceptual associations.

As explained previously, multiple occurrences of mappings of the same low-level physical dimension to underclasses of Spatialization (A17) were counted as a single occurrence, although all are referenced in Table 3. According to our classification method described in Section 3.2.3, independent low-level physical dimensions can be grouped together in the same intermediate-level conceptual dimension. Hence it is possible to identify two mappings of the same intermediate-level dimension to Spatialization, as in Projects 49, 50, and 52. Spatialization is the only auditory dimension belonging to the high-level category *Spatial* present among the fourteen most popular mappings. It is associated with the physical dimensions Location (P01) and Motion (P07), both belonging to the high-level category *Kinematics*, which supports Hypothesis 3.

It can be observed that more than half of the most popular associations between physical and auditory dimensions involve Pitch (A01), which supports Hypothesis 2.

All the other (i.e. not involving Pitch) mappings but one correspond to natural perceptual associations, which supports Hypothesis 1. The two mappings Location 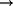 Spatialization and Motion 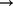 Spatialization can be easily understood: since Spatialization corresponds to the representation of a sound source in space and time, it corresponds to the natural representation of Location and Motion of sounding objects. The mapping Distance 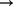 Loudness can be explained by the inverse distance law: the damping of sound waves in the transmission medium (e.g. air) leads to a decrease of sound intensity that is proportional to the distance to the sound source, and therefore to a decrease of loudness. The mappings Energy 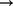 Loudness and Signal amplitude 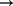 Loudness can be explained in that more energy dissipation leads to a larger amplitude of sound waves and therefore to an increase of loudness. It should be noted that these considerations take into account the polarity of the mapping, which was not studied in our systematic review. Further studies are required in order to verify this assumption.

The only mapping in Table 5 neither verifying Hypothesis 1 nor Hypothesis 2 is Density 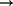 Duration.

#### 5.1.3 Use of auditory dimensions

In order to verify Hypothesis 2, we considered the frequency of use of auditory dimensions independently of the sonified physical quantities. The twelve most used auditory dimensions are presented in Table 6, together with their proportion of the total number of mapping occurrences. We performed pairwise Student's 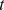-tests on this set of proportions in order to determine which auditory dimensions were used significantly more often than others (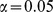). The third column in Table 6 shows how many auditory dimensions were used significantly less often than the one in the first column.

**Table 6:** Use of auditory dimensions regardless of the sonified physical dimensions.

Auditory dimension	Percentage of the total number of mappings	Number of auditory dimensions used significantly less often
Pitch	23.8	29 (100%)
Loudness	15.2	27
Duration	10.1	25
Spatialization	9.5	25
Tempo	5.9	21
Brightness	5.1	18
Timbre	3.6	14
Instrumentation	3.6	14
Spectral power	2.8	9
Spectral duration	2.4	5
Pitch range	2.0	3
Center frequency	2.0	3
of filter		

Most often used auditory dimensions regardless of the sonified physical dimensions. The second column corresponds to the percentage of the total number of mapping occurrences (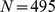) involving this auditory dimension. The third column indicates the number of auditory dimensions used significantly less often (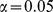). Pitch (A01) was found to be used significantly more often than all other 29 dimensions (A02 to A30) presented in Table 2.

It can be observed that Pitch (A01) is the most used auditory dimension in sonification mappings, and that it is significantly more often used than all other 29 auditory dimensions in our classification. This makes Hypothesis 2 verified for the set of publications included in the present systematic review. Other auditory dimensions often used are Loudness (A15), Duration (A20) and Spatialization (A17).

#### 5.1.4 Distribution of mappings: high-level trends

In order to examine significant discrepancies in the distribution of mapping occurrences, we performed statistical tests on the largest possible sample population by gathering physical and auditory dimensions into larger categories according to the classification presented in Section 3.2.3. The high-level categories corresponding to the classification of physical (respectively auditory) dimensions are presented in Table 1 (respectively Table 2). This has the advantage of reducing the subjective character of our classification, the five high-level categories being relatively stable and the inclusion of a particular mapping less subject to debate. On the other hand, the information sieve is probably too coarse for describing appropriately the design stage of a sonification system, which is more likely to involve intermediate or low-level dimensions. Distribution of mapping occurrences at an intermediate level will be examined in the next section.

Mapping occurrences were aggregated for all dimensions, for both physical and auditory domains, and summed over high-level categories. As previously, mappings referenced in Table 3 involving the multi-class dimension Spatialization (A17) were considered as a single mapping when corresponding to the same low-level physical dimension. Mappings of physical dimensions to subclasses of the multi-scale dimension Duration (A20) were considered as independent from each other and were therefore aggregated separately. In the case of a mapping of a given physical dimension to an auditory dimension belonging to two or more high-level categories, the mapping was counted once for each concerned high-level category. The resulting distribution of mapping occurrences is shown in Table 7.

**Table 7:** High-level trends in the distribution of mapping occurrences.

	Pitch-related		Loudness-related		Temporal		Timbral		Spatial	
	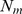	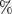	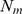	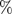	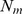	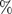	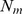	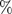	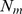	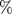
**Kinematics**	64	26.8	30	12.6	53	22.2	45	18.8	47	19.7
**Kinetics**	19	22.4	24	28.2	19	22.4	19	22.4	4	4.7
**Matter**	22	29.3	11	14.7	19	25.3	23	30.7	0	0.0
**Time**	17	25.0	8	11.8	17	25.0	24	35.3	2	2.9
**Dimensions**	20	32.3	6	9.7	15	24.2	18	29.0	3	4.8

Distribution of mapping occurrences aggregated in high-level categories for both physical and auditory domains. The number of mapping occurrences identified is reported (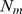) together with the corresponding proportion normalized against the high-level categories in the physical domain (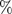).

**Figure 2:**
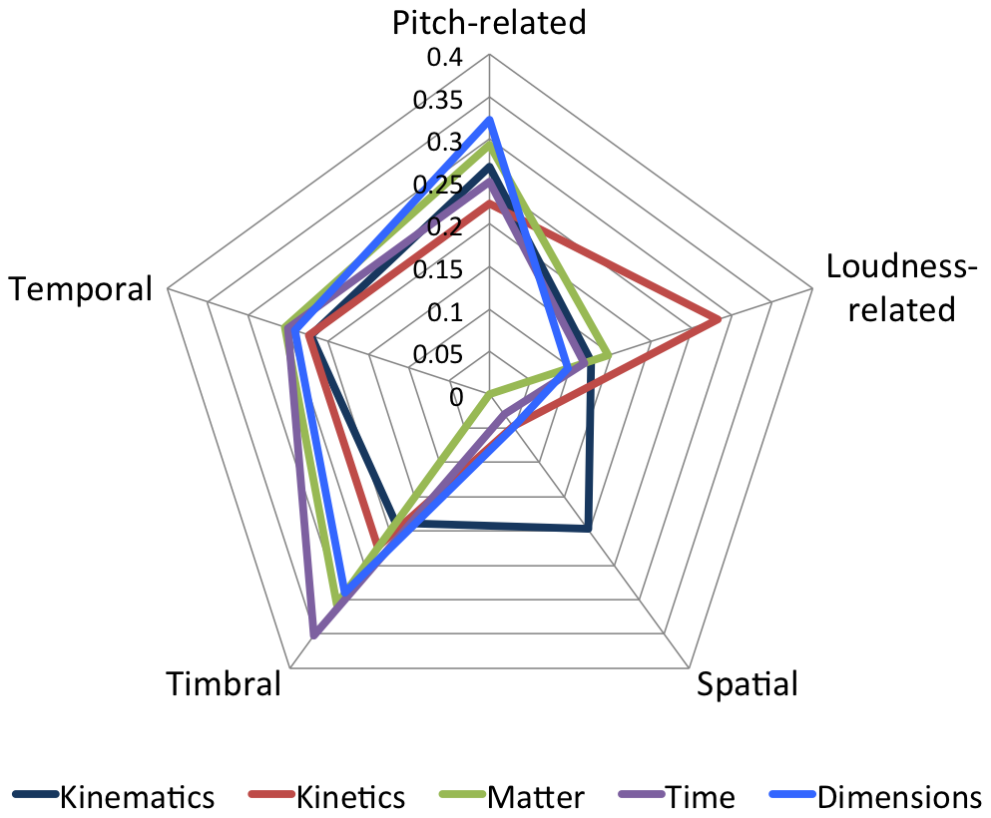
Proportions of mapping occurrences normalized against high-level categories in the physical domain. It can be observed that *Loudness-related* auditory dimensions are used mainly to sonify physical quantities belonging to the high-level category *Kinetics*. *Spatial* auditory dimensions are used mainly to sonify physical quantities belonging to the high-level category *Kinematics*.

Since we consider the choice of sonification mappings as a design problem, we set our focus on the typical issue for a sonification designer, i.e. establishing the type of auditory dimension to use in order to map a given physical dimension. To be able to compare mapping strategies for the different high-level categories in the physical domain, we normalized the data according to the number of mappings identified for these categories. That is, for each row corresponding to a high-level category in the physical domain, we computed the proportion of mapping occurrences corresponding to each high-level category in the auditory domain. These normalized proportions are presented in Table 7 and displayed in Figure 2.

Our objective is then to determine which categories in the auditory domain were used significantly more often for sonifying a specific high-level category of physical dimensions. For each high-level category in the physical domain, we performed pairwise Student's 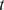-tests (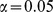) between high-level categories in the auditory domain on the normalized percentages. The following significant differences were observed:

For sonifying physical dimensions belonging to the high-level category *Kinematics*: *Pitch-related* and *Temporal* auditory dimensions were found to be used significantly more often than *Loudness-related* auditory dimensions.
For sonifying physical dimensions belonging to the high-level category *Kinetics*: *Spatial* auditory dimensions were found to be used significantly less often than auditory dimensions belonging to all other high-level categories (*Pitch-related*, *Loudness-related*, *Temporal*, and *Timbral*).
For sonifying physical dimensions belonging to the high-level category *Matter*: *Spatial* auditory dimensions were not used at all. All other high-level categories (*Pitch-related*, *Loudness-related*, *Temporal*, and *Timbral*) were used significantly more than 0%.
For sonifying physical dimensions belonging to the high-level category *Time*: *Timbral* auditory dimensions were found to be used significantly more often than *Loudness-related* auditory dimensions. *Spatial* auditory dimensions were found to be used significantly less often than auditory dimensions belonging to the high-level categories *Pitch-related*, *Temporal*, and *Timbral*.
For sonifying physical dimensions belonging to the high-level category *Dimensions*: *Loudness-related* auditory dimensions were found to be used significantly less often than auditory dimensions belonging to the high-level categories *Pitch-related*, and *Timbral*. *Spatial* auditory dimensions were found to be used significantly less often than auditory dimensions belonging to the high-level categories *Pitch-related*, *Temporal*, and *Timbral*.

Taking the dual approach, one could investigate the use of auditory dimensions in sonification works, i.e. what types of physical dimensions have been sonified using specific auditory dimensions. As explained above, we normalized the number of mapping occurrences in each high-level category in the physical domain against the total number of mapping occurrences identified in this category. The percentages obtained in this way are independent of the volume of projects implementing the sonification of specific physical dimensions, which allows us to compare the type of auditory dimensions used in the sonification depending on the high-level category of the physical input data. Our objective is then to determine which categories in the physical domain were sonified significantly more often using a given high-level category of auditory dimensions. For each high-level category in the auditory domain, we performed pairwise Student's 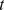-tests (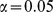) between high-level categories in the physical domain on the normalized percentages. The following significant differences were observed:

Using *Pitch-related* auditory dimensions: no significant differences were found between high-level categories in the physical domain.
Using *Loudness-related* auditory dimensions: physical dimensions belonging to the high-level category *Kinetics* were found to be sonified significantly more often than physical dimensions belonging to the high-level categories *Kinematics* and *Dimensions*.
Using *Temporal* auditory dimensions: no significant differences were found between high-level categories in the physical domain.
Using *Timbral* auditory dimensions: no significant differences were found between high-level categories in the physical domain.
Using *Spatial* auditory dimensions: physical dimensions belonging to the high-level category *Matter* were not used at all. Physical dimensions belonging to the high-level category *Kinematics* were found to be sonified significantly more often than physical dimensions belonging to all other high-level categories (*Kinetics*, *Time*, and *Dimensions*). This makes Hypothesis 3 verified for the set of publications included in the present systematic review.

It could be considered surprising not to observe an intrinsically natural association, namely sonifying physical dimensions belonging to the high-level category *Time* using *Temporal* auditory dimensions. Due to the temporal nature of sound, this association embodies a trivial case of mapping where input and output data share the same physical nature. The fact that this association was not highlighted by the present study can be explained by a sort of bias occurring in the identification process of the mappings making one of the most common mappings implicit. In fact, every project described as “interactive sonification” or “real-time sonification” (at least) could be seen as including a mapping from the dimension Instant — as physical input data — to the dimension Instant — as auditory output.

#### 5.1.5 Distribution of mappings: intermediate-level trends

While in the previous section we investigated trends in sonification design at a high level of description, it is often essential for sonification designers to make a clear distinction between intermediate-level dimensions within the same high-level category — e.g. by choosing between mapping Velocity (P02) and Acceleration (P03) to dissimilar auditory dimensions. These distinctions do not appear in the high-level classification, and finer trends may also level out when grouped together. Similarly, high-level categories in the auditory domain do not provide detailed information on the expected perceptual effects. For instance, mapping a given physical dimension to Allophone (A07) or Brightness (A12) can be perceived differently by the listener, leading to variable efficiency. On the other hand, low-level dimensions, being often very specific to the domain of application, would not allow us to identify statistically significant differences in the use of mappings. Intermediate-level dimensions presented in Tables 1 and 2 represent a more suitable level of description for attempting to set up design guidelines, or for investigating the use of sonification as in the present study.

The method used for identifying trends within high-level categories is not well suited to the relatively smaller number of mapping occurrences for each association between a physical dimension and an auditory dimension. In many cases, no occurrences at all of a particular mapping were found. The proportions of mapping occurrences can be obtained in the same manner as in the previous section, computing percentages normalized by the total number of mapping occurrences identified for each physical dimension. However, when performing pairwise Student's 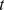-tests on these proportions, significant differences could only be obtained in very few cases. We chose to focus on the mappings having a proportion of use significantly greater than zero (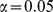). In Table 8 we present every physical dimension involved in at least one such mapping, together with the total number of identified mapping occurrences involving this dimension. In the third column we display the number of auditory dimensions having been used at least once to sonify this physical quantity. Finally, auditory dimensions used significantly more than 0% of the time are listed, together with the normalized percentage of use of the corresponding mapping. Physical dimensions not involved in such a mapping (i.e. for which no mapping was found to be used significantly more than 0% of the time) are not displayed in the table. In five cases, a particular auditory dimension was found to be used significantly more often than other auditory dimensions used at least once to sonify the same physical dimension (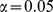). These cases are highlighted in Table 8.

**Table 8:** Intermediate-level trends in the distribution of mapping occurrences.

Physical dimension		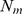	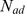	Auditory dimension		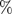
P01	Location	74	15	A17	Spatialization^*(28)^	32.4
				A01	Pitch^*(28)^	24.3
				A02	Pitch range	6.8
				A04	Instrumentation	5.4
				A20	Duration	5.4
				A21	Sequential position	5.4
P05	Distance	41	14	A15	Loudness^*(26)^	29.3
				A01	Pitch^*(23)^	22.0
				A20	Duration	9.8
P07	Motion	40	13	A01	Pitch	15.0
				A17	Spatialization	15.0
				A18	Doppler effect	12.5
				A15	Loudness	10.0
				A20	Duration	10.0
P16	Density	34	10	A01	Pitch^*(23)^	29.4
				A20	Duration	23.5
				A15	Loudness	11.8
P06	Orientation	26	11	A01	Pitch	26.9
				A17	Spatialization	19.2
P02	Velocity	25	8	A01	Pitch	24.0
				A19	Tempo	20.0
				A12	Brightness	16.0
				A15	Loudness	16.0
P30	Size	24	13	A01	Pitch	29.2
				A20	Duration	16.7
	Signal spectral	23	8	A01	Pitch	26.1
P28	energy			A08	Spectral power	21.7
	distribution			A15	Loudness	21.7
P13	Pressure	19	9	A01	Pitch	26.3
P08	Energy	15	8	A15	Loudness	40.0
P03	Acceleration	14	7	A01	Pitch	35.7
				A15	Loudness	28.6
P26	Event rate	14	6	A19	Tempo	28.6
P11	Temperature	12	7	A01	Pitch	41.7
P14	Signal amplitude	11	4	A15	Loudness	54.5
P27	Signal frequency	11	5	A01	Pitch	36.4
P23	Color luminosity	8	4	A15	Loudness	50.0
P17	Mass	6	3	A01	Pitch	66.7

For each intermediate-level physical dimension listed in the first column, the total number of mapping occurrences involving it (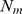) is displayed in the second column. The number of intermediate-level auditory dimensions that have been used at least once to sonify this physical dimension (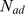) is shown in the third column, followed by the list of auditory dimensions used significantly more than 0% of the time, and by the corresponding proportion of use (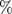). Auditory dimensions are marked with a star (*) whenever they have been found to be used significantly (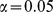) more often than other auditory dimensions used at least once to sonify the same physical dimension. The star is followed by the total number of auditory dimensions used significantly less often (including those not used at all).

#### 5.1.6 Example of multi-class dimension

In our classification, we introduced an example of multi-class auditory dimension by identifying different aspects (technical, theoretical, perceptual) of the implementation of Spatialization (A17). As explained previously, the classes defined in our classification correspond to these various aspects and are therefore not mutually exclusive (i.e., an occurrence of a mapping can belong to several classes simultaneously). An analysis of the distribution of mapping occurrences over these classes provides information about how sonification designers are implementing and using spatial sound. The proportion of mapping occurrences attached to each class relative to the total number of mapping occurrences involving Spatialization is presented in Table 9. It can be observed than Stereo panning (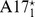), which can be considered as a very basic implementation of spatial sound, represents more than half of the uses of Spatialization. Pairwise Student's 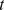-tests between the proportion attached to the different classes show that Stereo panning is used significantly (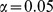) more often than all other classes.

**Table 9:** Use of auditory dimensions regardless of the sonified physical dimensions in the case of the multi-class dimension Spatialization.

Label	Class of spatialization	Proportion
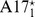	Stereo panning	53.
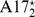	Multichannel panning	17.0
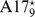	Non-specified spatialization method	14.9
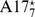	Interaural amplitude difference	12.8
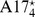	Head-related transfer function	10.6
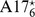	Interaural time difference	10.6
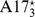	Vector base amplitude panning	6.4
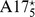	Ambisonics	6.4
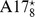	Interaural frequency difference	2.1

Classes of spatialization ranked according to their proportion of use with respect to the total number of mapping occurrences involving Spatialization (A17). Significantly higher percentages (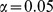) are indicated with a star (*).

We could potentially go further and conduct similar investigations as in Sections 5.1.4 and 5.1.5, in order to examine the dependency to the type of input physical dimensions of the distribution of mapping occurrences among classes of Spatialization. However, at the current stage of the study, these investigations would probably not provide conclusive results: at an intermediate level, the small number of occurrences identified for every distinct mapping makes the identification of marked trends unlikely. At a high level, it has been shown previously that *Spatial* auditory dimensions are used to sonify almost exclusively physical dimensions belonging to the high-level category *Kinematics*.

#### 5.1.7 Example of multi-scale dimension

We also provided an example of multi-scale dimension in our classification, namely Duration (A20), described in detail in Section 4.3. This auditory dimension was divided into three subclasses representing different time scales (rhythmic, event, ambient) and one subclass for cases where no time scale was specified. In a same way as in the multi-class example, a multi-scale structure allows to study this dimension at a higher level of detail by investigating the use of each scale as a separate dimension, either regardless of the input physical dimensions (as in Section 5.1.3) or depending on them (at a high level as in Section 5.1.4, or at an intermediate level as in Section 5.1.5). Unlike the classes from Spatialization (A17) presented in the previous subsection, the different scales of Duration are mutually exclusive by definition. Although considered as a separate dimension due to its belonging to an additional high-level category, we included the auditory dimension Spectral duration (A28) in the multi-scale analysis. In this way, we could consider the full range of durations by entirely reproducing Saue's classification of time scales [78].

As in the multi-class example, the small number of mapping occurrences in each category did not allow us to observe significant differences related to intermediate-level physical dimensions. In Table 10 we show the proportion for each scale normalized by the total number of mapping occurrences in each high-level physical category, as well as the proportion of mapping occurrences for each scale regardless of the physical dimension. Pairwise Student's 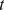-tests (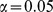) across high-level categories in the physical domain revealed no significant differences in the use of time scales. Pairwise Student's 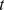-tests (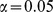) across the different scales showed that Rhythmic duration (A20_1_) was used significantly more often than all other scales when sonifying physical quantities belonging to the high-level category *Kinematics*, as well as regardless of the physical dimension.

**Table 10:** High-level trends in the case of the multi-scale dimension Duration.

	A28	A20_1_	A20_2_	A20_3_	A20_4_
**Kinematics**	19.0	66.7*	9.5	0.0	4.8
**Kinetics**	12.5	62.5	12.5	0.0	12.5
**Matter**	26.7	33.3	26.7	6.7	6.7
**Time**	11.1	55.6	11.1	0.0	22.2
**Dimension**	22.2	44.4	22.2	0.0	11.1
**Total**	19.4	53.2*	16.1	1.6	9.7

Proportions of mapping occurrences for each scale are shown aggregated in high-level categories in the physical domain, as well as regardless of the physical dimension (Total). The scales considered are: Spectral duration (A28), Rhythmic duration (A20_1_), Event duration (A20_2_), Ambient duration (A20_3_) and Non-specified duration scale (A20_4_). Significantly higher percentages (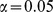) within a row are indicated with a star ().

#### 5.1.8 Assessed mappings

In order to gain maturity, the field of sonification requires sound evaluation methods to be developed and extensively used by the community. Recent review studies [42], [47] pointed out that evaluation of sonification systems is not systematic yet, although crucial from a design perspective. In most of the cases where some kind of evaluation is conducted, it consists either in a functional qualification of the sonification (i.e., showing that the display enables the execution of a given task) or in an assessment of its efficiency (i.e., investigating to which extent it has a valuable effect). As a consequence, the majority of these studies focus on the assessment of the auditory display as a whole, not investigating sonification mappings in detail, which means that mappings are often chosen in an ad hoc manner, or arbitrarily. The issue of mapping has only been tackled by few studies specifically focusing on psychoacoustical aspects, such as Projects 15 and 27 in the present systematic review.

As described in Section 3.2.2, we considered a mapping to be assessed as good (respectively assessed as bad) when it was found significantly more effective (respectively less effective) compared to other mappings based on objective tests. Mappings that were described as not functional were also assessed as bad. Assessment labels were assigned to a total of 30 mapping occurrences (15 were assessed as good, 15 as bad), representing 6.1% of the 495 mapping occurrences identified in the systematic review. All the involved mappings were assessed only once, with the exception of Velocity 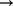 Tempo (P02 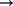 A19, assessed as good twice) and Motion 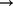 Rhythmic duration (P07 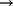 A20_1_, assessed as bad twice). Seven projects, representing 11.7% of the 60 projects considered in the systematic review, included at least one mapping occurrence with an assessment label. These rather small proportions highlight the general tendency in sonification works to set little focus on evaluation of individual mappings.

#### 5.1.9 Future mappings

Whenever a mapping was mentioned as a potentially interesting application but was not implemented in the framework of the project, it was assigned a special label (F). In total, 17 mapping occurrences were labeled as “future application”, representing 3.4% of the 495 mapping occurrences identified in the systematic review. Even if these particular mappings remained virtual, the researchers had expressed an advanced reflection on the sonification design. For this reason, we decided not to distinguish these particular mapping occurrences from normal occurrences (i.e. those actually implemented) when performing the statistical tests presented previously.

#### 5.1.10 Keyword-based analysis

Beyond the classification into conceptual intermediate-level dimensions and the grouping into high-level categories introduced previously, it is possible to apply various filters to the low-level dimensions in order to look for specific information. As an example, we filtered the low-level physical dimensions according to two complementary keywords: Horizontal and Vertical. In the following we present the low-level dimensions included in the category formed by each keyword. Each of them belongs to an intermediate-level dimension in our original classification, which is specified via its label.

For the keyword Horizontal: horizontal position (P01), x position (P01), map: longitude (P01), azimuth angle (P06), radial direction (P06), horizontal direction (P07), horizontal movement of mouth corners (P07), width (P32), texton width (P32).
For the keyword Vertical: vertical position (P01), y position (P01), vertical location of a maximum (P01), map: latitude (P01), slope (P06), vertical displacement (P07), vertical movement of lips (P07), vertical direction (P07), vertical displacement deviation magnitude (P07), vertical force (P10), height (P32), map: altitude (P32), altitude deviation (P32).

The same statistical tests as those performed in the previous subsections can be performed on keyword-based categories. For the sake of illustration, we investigated intermediary-level trends for the two categories corresponding to the keywords in the same manner as in Section 5.1.5.

Normalized proportions of mapping occurrences were computed. Mappings used significantly more than 0% of the time (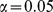) are shown in Table 11. For each keyword-based category, the total number of mapping occurrences identified is presented together with the number of auditory dimensions having been used at least once. Finally, auditory dimensions used significantly more than 0% of the time are listed, together with the normalized percentage of use of the corresponding mapping. Cases where an auditory dimension was found to be used significantly more often than other auditory dimensions used at least once to sonify the same keyword-based category (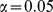) are highlighted in the table. We can observe that physical dimensions related to horizontality are most often sonified through Spatialization, while those related to verticality are most often sonified via changes in Pitch. This particular trend was not visible in the original classification due to the grouping of low-level physical dimensions belonging to the two keyword-based categories in different intermediate-level dimensions.

**Table 11:** Intermediate-level trends in the case of the keyword-based categories Horizontal and Vertical.

Physical dimension	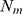	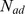	Auditory dimension used significantly more than 0% of the time	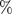
Horizontal	22	14	Spatialization^*(27)^	36.4
Vertical	30	9	Pitch^*(28)^	46.7
			Loudness	13.3

For each keyword-based category, the total number of mapping occurrences (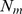) is displayed, followed by the number of intermediate-level auditory dimensions that have been used at least once to sonify this category (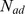). The list of auditory dimensions used significantly more than 0% of the time is shown in the third column, followed by the corresponding proportion of use (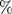). Auditory dimensions are marked with a star (*) whenever they have been found to be used significantly (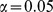) more often than other auditory dimensions used at least once to sonify the same keyword-based category. The star is followed by the total number of auditory dimensions used significantly less often (including those not used at all).

Other interesting trends could be revealed by filtering physical or auditory data dimensions using carefully selected keywords. For instance, we could build up categories gathering low-level physical dimensions related to Uncertainty, e.g. including dimensions such as the deviation of various physical quantities from a reference value. We could also consider a specific domain of application, e.g. the sonification of EEG by defining a category gathering all low-level dimensions originating from that domain.

### 5.2 Other trends

In the previous subsections we focused on mapping frequencies in order to investigate associations between physical and auditory dimensions that have been used in past sonification works. Different approaches can be taken to extract other type of information from the sixty projects we have analyzed.

#### 5.2.1 Project-related trends

Instead of taking a mapping-centered approach as in Section 5.1, we can investigate project-related trends. The same type of statistical tests can be performed, considering the proportion of projects using a specific mapping or dimension. For the sake of illustration, we investigated the use of auditory dimensions throughout the sixty projects included in this study. In Table 12, we present the proportion of projects using specific auditory dimensions at least once. The eight auditory dimensions used by the largest number of projects are shown in the table. We performed pairwise Student's 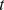-tests on this set of proportions in order to determine which auditory dimensions were used by significantly more projects than others (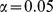). The third column in Table 12 shows how many auditory dimensions were used by significantly fewer projects than the one in the first column.

**Table 12:** Project-related trends: use of auditory dimensions regardless of the sonified physical dimensions.

Auditory dimension	Percentage of projects using the dimension at least once	Number of auditory dimensions used by significantly fewer projects
Pitch	86.7	28
Loudness	73.3	27
Spatialization	51.7	26
Duration	40.0	24
Brightness	23.3	13
Timbre	20.0	10
Tempo	16.7	6
Spectral power	15.0	6

Percentage of projects using specific auditory dimensions at least once. The eight auditory dimensions used by the largest number of projects are displayed. The third column indicates the number of other auditory dimensions used significantly less often (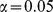).

The same approach can be taken in order to investigate the proportion of projects sonifying given physical dimensions, or using specific associations between categories — both at an intermediate and at a high level.

#### 5.2.2 Historical distribution

**Figure 3:**
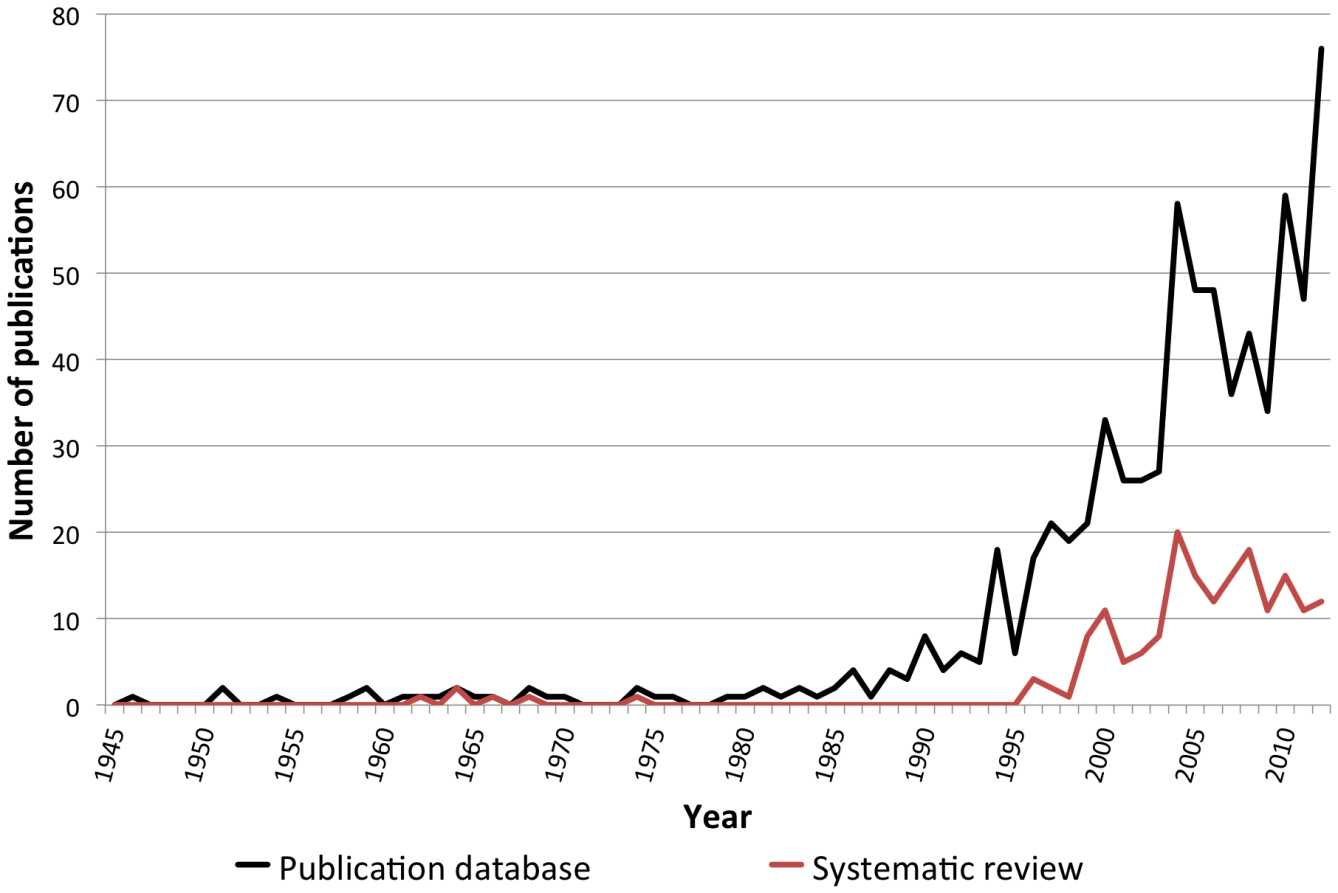
Historical distribution of sonification works according to the year of publication. The red curve corresponds to the publications considered for the present systematic review. The black curve corresponds to the works included in the publication database, including those considered for the present systematic review.

We considered the distribution over the time of sonification works from the publication database, according to the year of publication. Publications included in the present study should be distinguished from remaining entries: while the former correspond to practical applications of sonification of physical quantities, the latter are only considered as potentially interesting at this stage, and will be included in future developments of the systematic review provided that they match the criterion for inclusion defined in Section 3.1.1. The historical distribution of database entries — comprising both included and remaining publications — is displayed in Figure 3, together with the distribution of included works alone. The earliest entry in the database is a technical report published in 1946. The distribution of database entries is sparse until the 1980s, then shows a slow growth of the number of publications until the beginning of the 1990s, followed by an irregular but rapid increase since then.

The historical component of a mapping could be studied as well in the future by monitoring the evolution of its use over the time. This could be a way of assessing the degree of success of a mapping.

#### 5.2.3 Project classification

**Figure 4:**
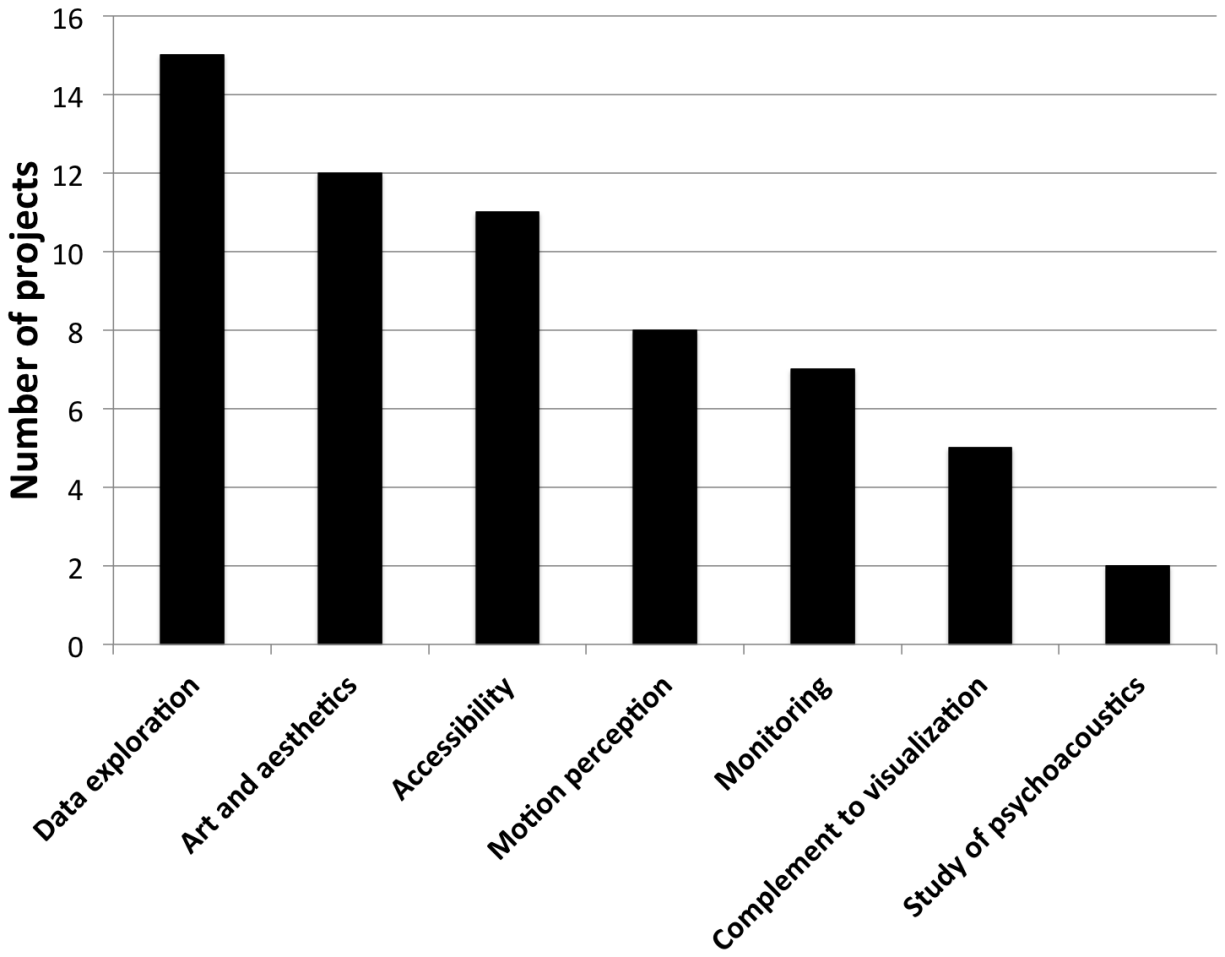
Distribution of the projects considered in the present systematic review classified according to their primary function.

The sixty projects included in the present systematic review represent a sample of typical sonification works, and can be used to initiate a function-based classification for applications of sonification. Relating the function of a sonification project to its utilization of characteristics of sonification defined in Section 1.2, we defined seven broad categories encompassing these characteristics: *monitoring*, *motion perception* (including kinesthesia, training, and rehabilitation), *accessibility* (including sensory substitution, and mobility aid), *data exploration* (including data mining), *complement to visualization* (including sonification of maps), *art and aesthetics*, and *study of psychoacoustics*. All projects were classified according to their function as expressed by the researchers. We chose to consider only the primary function of a given project, although secondary functions were also described in many cases. For instance, Project 58 corresponds to an art installation sonifying the trajectory of cosmic particles. It belongs to the category *art and aesthetics* in our classification, but also represents a kind of *motion perception*, which was considered as a secondary function and is therefore not reported here. The resulting classification is presented in Figure 4.

Not surprisingly (because corresponding to one of the criteria for inclusion in the systematic review), most of the projects are associated with categories corresponding to a practical function: *data exploration*, *accessibility*, *motion perception*, and *monitoring*. Artistic works represent 20% of the projects, which is a relatively large part considering that the artistic nature of sonification is disputed. Only 2 projects out of 60 correspond to studies of psychoacoustics aiming at assessing perceptual effects of sonification mappings. This example of classification is based on a limited sample of projects, employs rather broad categories, and takes into account only the primary function of the projects. More advanced ways of classifying sonification projects could be studied in the future. Other sorts of project classification could be conducted, e.g. according to the discipline attached to the sonified data.

#### 5.2.4 Sonic material

The sixty projects of the present systematic review also provide information about the types of sonic material used to implement sonification applications. The choice of sonic material is critical for sonification design, insofar as it can dramatically affect the efficiency of specific mappings, or even of the entire auditory display.

**Figure 5:**
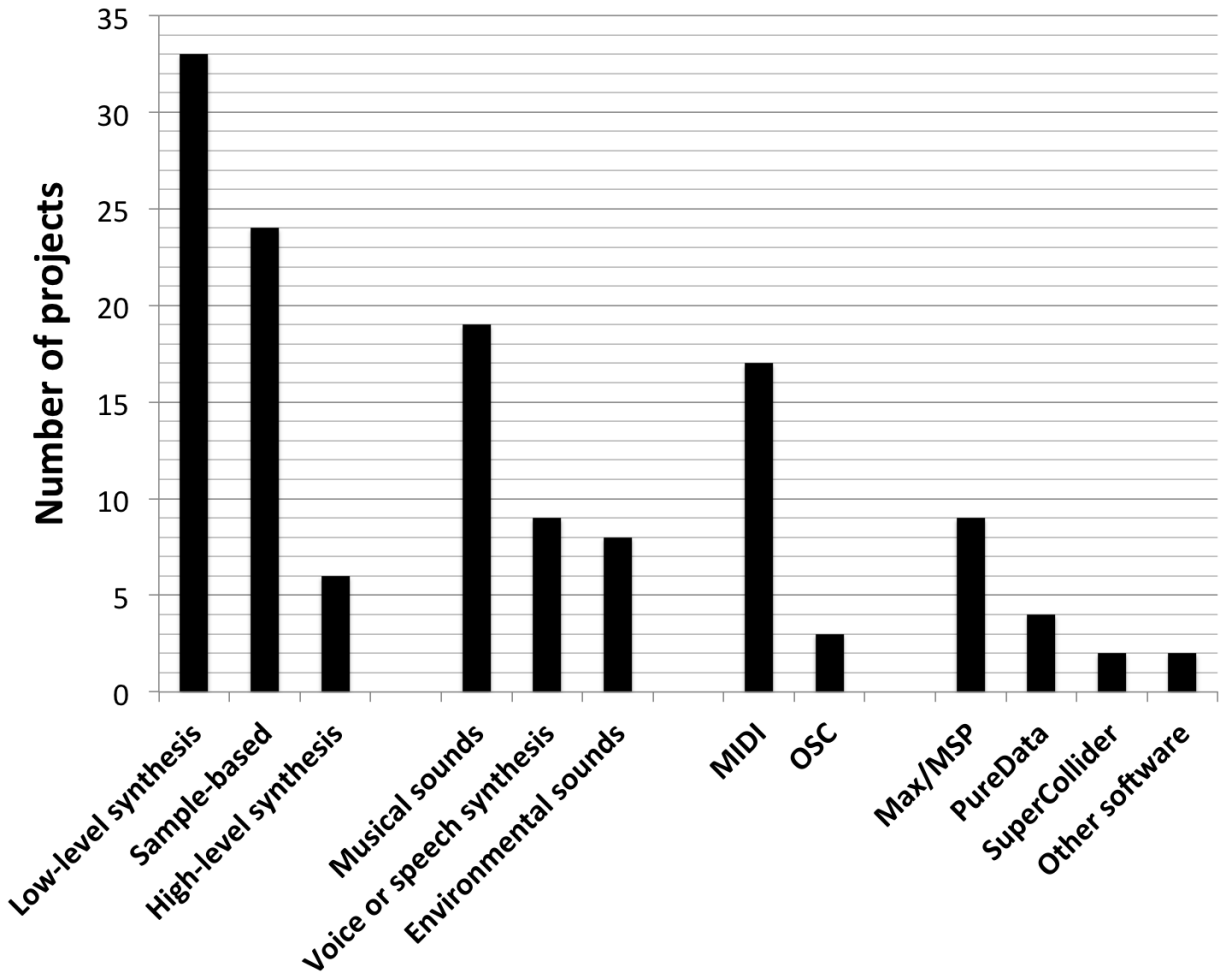
Sonic material used in the projects considered in the present systematic review. Results are presented in groups corresponding to level of synthesis, general category of sound, standard protocols, and software.

For each project, a detailed description of the sonic material is given in the fourth column of Table 3. Several perspectives can be taken to describe the sonic material, among which the level of synthesis, the general category of sound, existing standard protocols, and the software that was used. Three different levels of synthesis were found among the projects: *low-level synthesis*, *high-level synthesis*, and *sample-based displays*. Low-level synthesis corresponds to cases where the auditory display is constituted by a waveform resulting from direct production and processing of a signal (e.g. pure tones, FM synthesis, filtered noise), whereas high-level synthesis corresponds to the use of more advanced pre-existing models (e.g. models for voice synthesis or physical interactions). Sample-based displays are formed by pre-recorded sound files that are played back, and optionally processed simultaneously. Three general categories of sounds were identified: *musical sounds*, *voice or speech synthesis*, and *environmental sounds*. Two standard protocols for information communication were used: *MIDI* and *OSC*. Finally, we investigated the use of several common software platforms for sound design and production. In Figure 5 we show the number of projects associated with each category.

The issue of the sonic material used in sonification applications was recently addressed in the review study by Bearman and Brown [43]. Investigating the use of different “*synthesis tools”*, they found that the most popular software platforms were SuperCollider and PureData.

## Conclusions and Perspectives

In this article we conducted a systematic review of sonification of physical quantities. The first step was to build up a database of publications related to practical applications of sonification, currently comprising 739 entries. Several aspects of this database were investigated: we presented the historical distribution of the entries of the database, providing a picture of the field of sonification since 1945. The publication database, constituting a resource for sonification researchers, could be extended in the future to include work dealing with audification, considered as a direct mapping of any physical dimension to instantaneous sound pressure. Theoretical works and projects involving sonification of more abstract (non-physical) data such as price or web traffic flow could also be incorporated. From the publication database, we selected randomly sixty sonification projects for the systematic review, corresponding to a total of 179 scientific publications. These projects constitute a sample of typical sonification works, and were classified according to their primary function. The sonic material used in the projects was analyzed from different perspectives such as the level of synthesis, the nature of the sound, and the software platform used.

We introduced a method for classifying mappings extracted from sonification projects. A list of conceptual dimensions was drawn up for both sonified physical quantities and auditory dimensions used to render auditory displays. These conceptual dimensions were obtained taking a bottom-up approach; therefore, the list of physical dimensions depends on the domain of sonification mappings, i.e. the nature of the data that was sonified in the selected projects. This list will evolve gradually when additional projects are included in the analysis. On the other hand, the list of auditory dimensions, obtained by the same bottom-up approach, has reached a relatively stable state, due to the fact that the codomain of sonification mappings is always the same, namely the auditory domain. However, sharper focus can be given to specific auditory dimensions of interest through a separation in different scales or different classes. We also provided an example of multi-class dimension (Spatialization, A17), and one of multi-scale dimension (Duration, A20).

For each project, associations between physical and auditory dimensions were identified, constituting a database of sonification mappings. A total number of 495 mapping occurrences were identified. Additional information was attached to mappings in this database whenever their efficiency was assessed (as good or as bad), or if they were mentioned as interesting future development but not implemented at the time of publication. We have found that only a marginal proportion of mapping occurrences have been assessed, highlighting the lack of evaluation in sonification design. An analysis of the frequency of use of mappings was performed at the level of the conceptual dimensions previously described, as well as for high-level categories gathering these dimensions for both physical and auditory domains. This analysis confirmed the following prior hypotheses: Pitch is by far the most used auditory dimension in sonification mappings, and *Spatial* auditory dimensions are almost exclusively used to sonify *Kinematic* physical quantities. Results were found consistent with the following third hypothesis: the most popular mappings follow the logic of ecological perception. The most often used mappings not involving Pitch correspond indeed to natural perceptual associations. Nevertheless, the polarity of the involved mappings should be investigated in order to be able to demonstrate this hypothesis. By normalizing the number of mapping occurrences against the total number of mapping occurrences identified for a given physical dimension, we could determine the most popular mappings independently of the domain of application. Being often used does not demonstrate that these mappings are the most efficient ones, but it suggests to investigate them first, when developing future guidelines for sonification design, both by examining their polarity and by assessing them, e.g. with help of psychophysical tests.

### 6.1 Characterization of sonification via mappings

The concept of mapping is central in the “working definition” of sonification by Scaletti reported in Section 1.1, but did not appear in many of the later definitions. Throughout the reading process conducted in the framework of the present systematic review, we found its role significant when considering the potential inclusion of a given work in the analysis. When designing criteria for inclusion in the publication database prior to the reading process, as described in Section 3.1.1, we already considered the possibility to extract at least one mapping as being a qualifying factor. In fact, it proved to be a necessary and sufficient condition for a publication to be included in the analysis: all works that were included contain at least one description of a sonification mapping from a physical dimension to an auditory dimension, and any work that contains such a mapping was considered as being a relevant sonification application. Because we were interested in sonification of physical quantities, we identified the domain of mappings with the physical domain. Considering the possibility to extract at least one such mapping as a necessary and sufficient condition for inclusion, we developed de facto a characterization of sonification of physical quantities.

This way of characterizing a subdomain of sonification can be extended to characterize sonification itself, considering carefully the domain and the codomain of mappings. The various specificities of the nature of sonification presented in Section 1.1 can be expressed in line with this approach, e.g. by imposing restrictions on the domain and on the codomain. For instance, a part of the definition such as “*the use of nonspeech audio”* is ambiguous and might be interpreted erroneously as an exclusion of voice and speech synthesis in the sonic material used in the sonification. Using mappings to characterize this aspect amounts to restrict the codomain of mappings by excluding the semantics attached to speech. The purpose of sonification — to communicate information — is embedded in the condition that the mappings have to be a conscious design choice to be taken into account. This characterization process does not enable the distinction between scientific and artistic works, but the need for such a distinction is questionable. Further theoretical considerations are required to build up a robust characterization process, but we believe that an approach centered on mappings could constitute a good basis for a new definition of sonification.

## Supporting Information

Checklist S1 **PRISMA checklist.**
(PDF)

Flow Diagram S1 **PRISMA flow diagram.**
(PDF)
