# Correction: A Systematic Review of Mapping Strategies for the Sonification of Physical Quantities

**DOI:** 10.1371/journal.pone.0096018

**Published:** 2014-04-23

**Authors:** The PLOS ONE Staff

Table 9 has been corrected.

**Table 9:** Use of auditory dimensions regardless of the sonified physical dimensions in the case of the multi-class dimension Spatialization.

Label	Class of spatialization	Proportion
A17 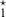	Stereo panning	53.2*
A17 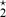	Multichannel panning	17.0
A17 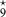	Non-specified spatialization method	14.9
A17 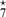	Interaural amplitude difference	12.8
A17 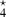	Head-related transfer function	10.6
A17 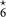	Interaural time difference	10.6
A17 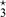	Vector base amplitude panning	6.4
A17 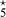	Ambisonics	6.4
A17 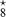	Interaural frequency difference	2.1

Classes of spatialization ranked according to their proportion of use with respect to the total number of mapping occurrences involving Spatialization (A17). Significantly higher percentages () are indicated with a star (*).

Please see the corrected Table 9 here:
