# Supplementary material for: A Systematic Review of Mapping Strategies for the Sonification of Physical Quantities
Source: PLoS One. 2013 Dec 17;8(12):e82491. doi: 10.1371/journal.pone.0082491 (PMC3866150; doi:10.1371/journal.pone.0082491)
Supplement: Flow Diagram S1 — PRISMA flow diagram. (PDF) [file pone.0082491.s002.pdf]

PRISMA 2009 Flow Diagram

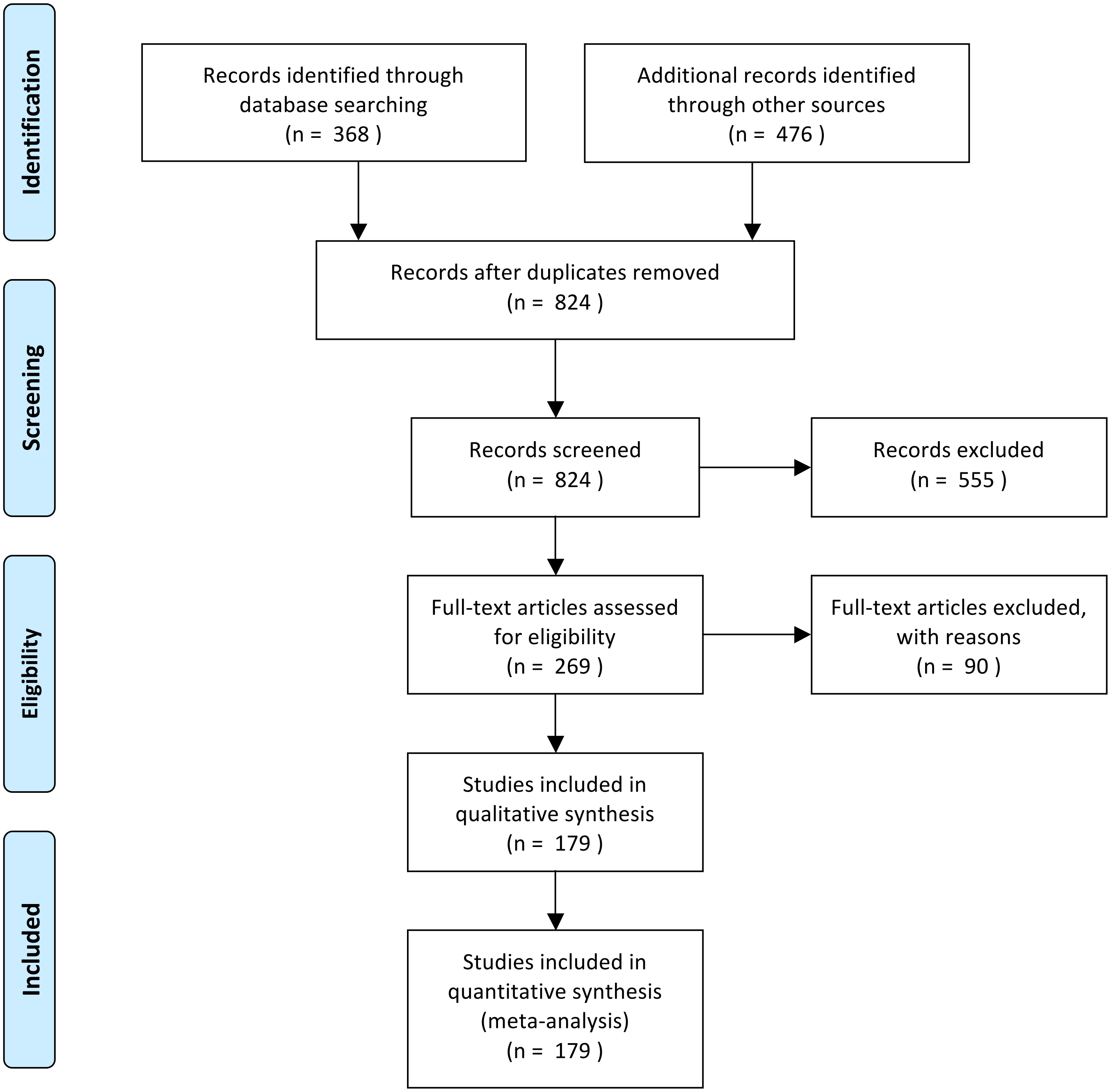

From: Moher D, Liberati A, Tetzlaff J, Altman DG, The PRISMA Group (2009). *P*referred *R*eporting *I*tems for *S*ystematic Reviews and *M*eta-*A*nalyses: The PRISMA Statement. PLoS Med 6(6): e1000097. doi:10.1371/journal.pmed1000097

For more information, visit www.prisma-statement.org.
